# Pangenomics of the death cap mushroom *Amanita phalloides*, and of Agaricales, reveals dynamic evolution of toxin genes in an invasive range

**DOI:** 10.1038/s41396-023-01432-x

**Published:** 2023-05-23

**Authors:** Milton T Drott, Sung Chul Park, Yen-wen Wang, Evan Harrow, Nancy P Keller, Anne Pringle

**Affiliations:** Present address: USDA-ARS Cereal Disease Laboratory, St. Paul, MN, USA; 1 Department of Medical Microbiology and Immunology, Department of Bacteriology, University of Wisconsin, Madison, WI, USA; 2 Departments of Botany and Bacteriology, University of Wisconsin, Madison, WI, USA

## Abstract

The poisonous European mushroom *Amanita phalloides* (the “death cap”) is invading California. Whether the death caps’ toxic secondary metabolites are evolving as it invades is unknown. We developed a bioinformatic pipeline to identify the MSDIN genes underpinning toxicity and probed 88 death cap genomes from an invasive Californian population and from the European range, discovering a previously unsuspected diversity of MSDINs made up of both core and accessory elements. Each death cap individual possesses a unique suite of MSDINs, and toxin genes are significantly differentiated between Californian and European samples. MSDIN genes are maintained by strong natural selection, and chemical profiling confirms MSDIN genes are expressed and result in distinct phenotypes; our chemical profiling also identified a new MSDIN peptide. Toxin genes are physically clustered within genomes. We contextualize our discoveries by probing for MSDINs in genomes from across the order Agaricales, revealing MSDIN diversity originated in independent gene family expansions among genera. We also report the discovery of an MSDIN in an *Amanita* outside the “lethal *Amanitas*” clade. Finally, the identification of an MSDIN gene and its associated processing gene (*POPB*) in *Clavaria fumosa* suggest the origin of MSDINs is older than previously suspected. The dynamic evolution of MSDINs underscores their potential to mediate ecological interactions, implicating MSDINs in the ongoing invasion. Our data change the understanding of the evolutionary history of poisonous mushrooms, emphasizing striking parallels to convergently evolved animal toxins. Our pipeline provides a roadmap for exploring secondary metabolites in other basidiomycetes and will enable drug prospecting.

## Introduction

An enormous scientific literature characterizes the ecological and evolutionary mechanisms enabling the success of organisms introduced to new environments [1, 2]. Research has been used to guide mitigation of the ecological and economic damage resulting from biologic invasions [3]. However, invasion biology has focused primarily on plant [4] and animal [5] species. Gladieux et al. [6] speculate that fungal invasions may be more common than plant or animal invasions, suggesting the inconspicuous nature of fungi stymies research. Meanwhile, invasive fungi have devastated forests [7], driven several amphibians and bats to near extinction [8, 9], and cause human disease [10]. But relatively little is known about the traits enabling the success of invasive nonpathogenic (mutualistic and decomposer) fungi in new environments.

Fungal secondary metabolites (SMs), as distinct from primary metabolites, appear to mediate ecological interactions [11]. SMs are common in many fungi and shape the niches of species by mediating competition [12–14], influencing host range [15, 16], and protecting against environmental stressors [17–19]. Until recently SM profiles were thought to define species, with relatively little or no variation within a species, but new data suggest local adaptations may manifest in population-specific patterns of intraspecific SM diversity [20]. Population-specific SMs influence the geographic ranges of species and inform macroevolutionary inferences [20]. Most research on fungal SMs targets ascomycetes. While mushrooms are notorious for their chemistry, particularly their abilities to cause hallucinations and poisonings, the complex life histories, genetics, and technical challenges of manipulating basidiomycetes have precluded the development of tools to catalog mushroom SM diversity and limited descriptions of their evolutionary history.

The “death cap” *Amanita phalloides* (Vaill. ex Fr.) Link is an infamous, poisonous ectomycorrhizal basidiomycete native to Europe and introduced elsewhere, including to North America and in particular, California [21, 22]. Ectomycorrhizal fungi can shift competitive dynamics between plant species [23], alter soil community structure [24], facilitate metal homeostasis [25], and influence nutrient cycling [26]. The potential ecological consequences of the death cap’s range expansion in California remain unknown, but its abundance [27] and the often-fatal poisonings associated with its mushroom [28] lead many authors to identify it as invasive [29]. Factors contributing to the spread and success of non-native ectomycorrhizal fungi have been identified based on models of primary succession [30] but whether competitive or defensive interactions with native biodiversity also facilitate spread remains uninvestigated. Interspecific interactions are often mediated by SMs; in plants, SMs can have particularly pronounced effects in a species’ invasive range, offering “novel weapons” when competing with “naïve” native organisms [31, 32]. Whether different populations of *A. phalloides* use or exploit weapons is unknown.

A few of the compounds underpinning *A. phalloides’* notorious toxicity are identified, including α-amanitin, phalloidin, and phallacidin [33]. These toxins are constituents of a recently discovered class of SM named as “MSDIN”, based on the conserved amino acid “leader” motif Met-Ser-Asp-Ile-Asn. MSDIN genes encode short (35–36 amino acid), ribosomally synthesized and post-translationally modified peptides (RiPPs) [34]. A MSDIN-specific prolyl oligopeptidase (POPB) cleaves the conserved leader and “follower” portions of the MSDIN pro-protein, resulting in a cyclized “core” that becomes or is the final product [35]. While the cyclization of diverse MSDIN core peptides has been demonstrated through chemical analysis (e.g., Zhou et al. [36]), cyclization is often inferred directly from sequence data based on the highly conserved leader and follower motifs. Emblematic of the SM literature, research into MSDINs has focused on identifying new sequences from different species using a single or small number of reference genomes; little is known about diversity of these genes within species. To date we know that the MSDIN gene encoding α-amanitin is discontinuously spread across Agaricales, found only in the genera *Galerina*, *Lepiota*, and *Amanita* [33]. Several authors infer horizontal gene transfer to explain the discontinuous distribution of MSDIN genes [33, 37]. However, questions remain about the relative importance of both horizontal and vertical transmission in the genes’ evolutionary history (reviewed by Walton [33]). Recent identification of additional genes involved in the biosynthesis of α-amanitin suggests a different, as-yet-unidentified ancestral species may also be involved in the origins of MSDINs [38]. While α-amanitin is the only MSDIN produced by *Galerina* species [39, 40], the MSDIN gene family has expanded in the genus *Amanita*, resulting in dozens of unique MSDINs found in different species [34, 41–43]. MSDINs are hypothesized to mediate defense against insects, nematodes, and other animals [33]. However, a dearth of information about the intraspecific diversity of these compounds precludes the targeted ecological experimentation needed to explain the natural history and diversification of MSDINs in *Amanita*.

We sought to establish the potential for MSDIN gene products to shape interactions in an invasive California (CA) population by developing a bioinformatics pipeline to automate identification of MSDIN genes, specifically asking: (1) Do the genomes of Californian *A. phalloides* individuals each encode the same suite of MSDINS, if not, how is the MSDIN pangenome structured and are there core and accessory genes? (2) Are toxin genes maintained by natural selection? (3) Does the MSDIN gene family expansion predate speciation of *Amanita* spp. and how did the expansion shape the physical distribution of MSDINs within genomes? We also used chemical analyses to confirm the production of well-known MSDIN products and to look for new MSDIN peptides, emphasizing that MSDIN gene products are expressed and translated, resulting in measurable phenotypes and compounds with the potential to mediate ecological interactions.

## Methods

### Mushroom collecting, genome sequencing, assembly, and SNP variant calling

Mushrooms of *A. phalloides* were intensively sampled from a single invasive population at Point Reyes National Seashore, CA, USA (*n* = 68), and from native populations across Europe (*n* = 20), for a total of 88 genomes (Table S1). Mushrooms were collected between 1978 and 2015, with most sampling concentrated in 2014 and 2015 (Table S1). We also collected mushrooms putatively identified as *Amanita thiersii* and *Amanita foetens* in Kansas (*A. thiersii*) and Argentina (*A. foetens*), and we used these genomes in comparative analyses and as controls. We numbered collected mushrooms as 1–90 (e.g., 1mAP, 2mAP). Our numbers correspond to the AmanitaBASE numbers used in other publications generated from the Pringle laboratory [44] (Table S1). A single mushroom (7mAP; Table S1) with a very poor-quality assembly was used in initial scans of MSDIN genes, but it was removed from subsequent analyses (the 7mAP assembly was 10% complete as measured by BUSCOs, and the next poorest assemblies of *A. phalloides* were 72.1, 91 and 92% complete, respectively).

**Fig. 1:**
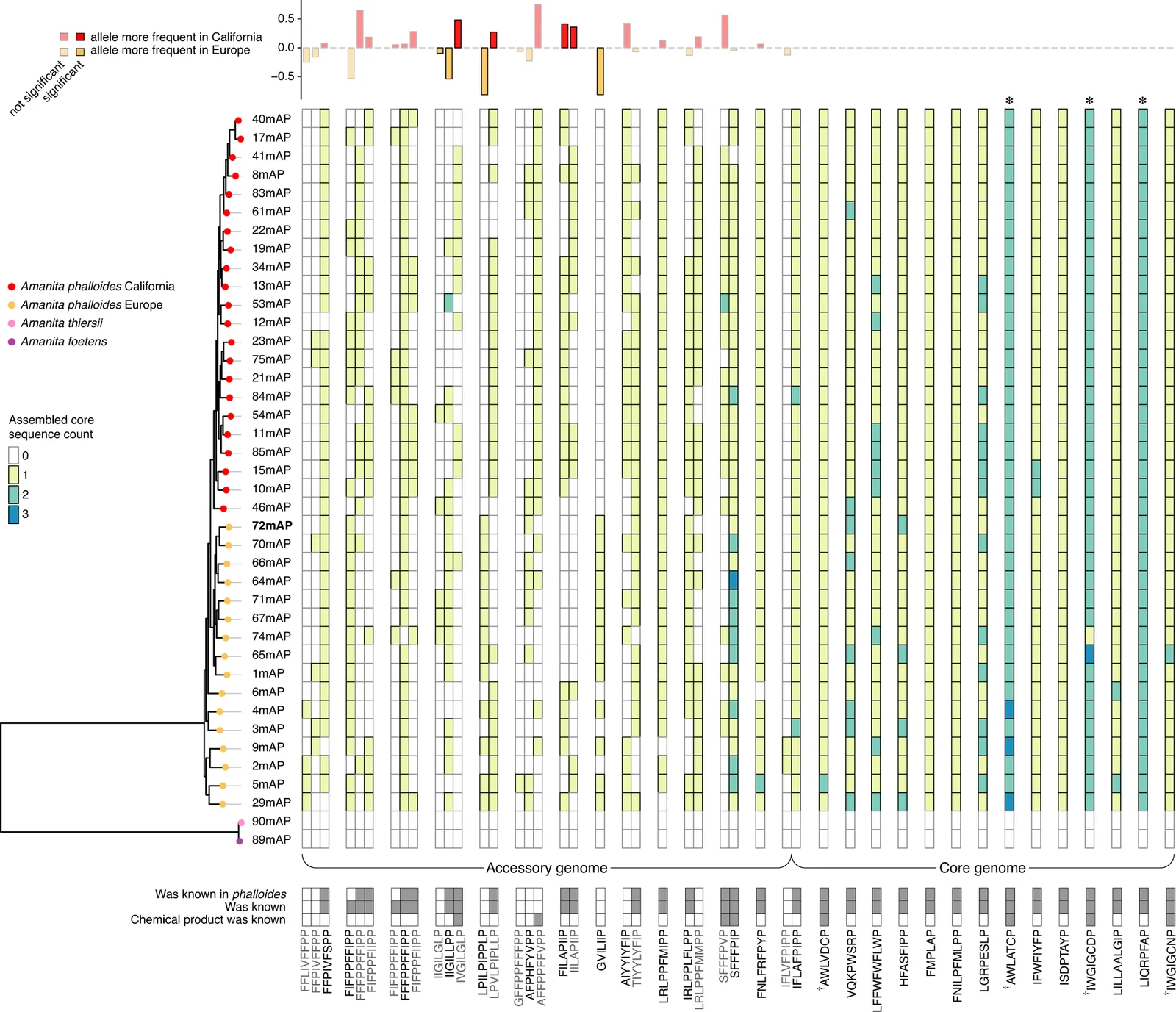
Heatmap of the MSDINs found in a clone-corrected set of *Amanita* spp. genomes. Phylogenetic relationships among 38 *A. phalloides*, one *Amanita thiersii* and one *Amanita foetens* specimens are depicted in a maximum-likelihood tree constructed using genome-wide SNPs. Colored boxes indicate the presence of an MSDIN allele as defined by a specific “core” sequence. Loci representing alleles, or groups of alleles, at a specific physical location in a genome are separated by blank columns. Counts greater than one are indicative of duplicated loci encoding identical alleles. In the high-quality reference genome, we validated three duplications (see Supplementary Methods), but we were unable to validate duplications in other genomes and assembly errors are possible. For this reason, we collapsed the three duplicated loci from the reference genome (*) to provide congruence with other putative (but unresolved) duplications. If a core sequence was inferred from SNP data, its count value was set to one even if it was not found in the genome assembly. Determination of “knowns” at the bottom of the figure is based on previous reports of MSDIN core sequences or characterization of chemical products. Locus names (in black text) are based on the allele present in the reference genome assembly (see bolded 72mAP). The name of a single locus not found in the reference genome (FILAPIIP) was chosen alphabetically. The panel above the heatmap indicates the differences in allele frequencies between Californian (in red) and European (in orange) individuals. Significant differences in allele frequencies at each locus are based on Analysis of Molecular Variance. Loci AFPHFYVPP, FFPIVFSPP, FIFPPFFIPP and SFFFPIP were not included allele frequency analyses because of uncertainty in some calls (see Results). ^†^ These MSDIN sequences are associated with mature products with characterized bioactivity as toxins. From left to right: phallacidin (AWLVDCP), phalloidin (AWLATCP), β-amanitin (IWGIGCDP) and α-amanitin (IWGIGCNP).

Genomic DNAs were extracted and sequenced using an HiSeq2500 (Illumina) platform in Rapid mode with 251 bp paired end reads as described by Wang et al. [45]. A single isolate (72mAP) was also sequenced with a PacBio RS II Sequel platform for long reads (N50 = 6310 bp) and data used to assemble the reference genome [45]. SNPs were called (Wang et al. [45]) using the GATK pipeline [46] (Table S1), following best practices (https://gatk.broadinstitute.org/hc/en-us/articles/360035535932-Germline-short-variant-discovery-SNPs-Indels-). SNPs were then hard filtered using parameters defined in the GATK workflow for non-model organisms (https://gatk.broadinstitute.org/hc/en-us/articles/360035890471), specifically: QD < 2.0 || FS > 60.0 || MQ < 40.0|| MQRankSum < −12.5 || ReadPosRankSum < −8.0 for SNPs; and QD < 2.0 || FS > 200.0 || ReadPosRankSum < −20.0 for indels. Mushrooms belonging to the same genet (clones produced by a single mycelial individual) were identified as described in Wang et al. [45]. In brief: clones were identified using two different approaches, the first involving a Euclidean distance matrix resulting from filtered SNPs and the second based in kinship analyses (Wang et al. [45]). Additionally, a single isolate (1mAP) processed in past work, but not used because of concerns for low-quality sequence (that were not evident in our efforts), was clone corrected based on phylogenetic relationships identified here (Fig. 1). Both approaches identified the same genetic individuals, or genets [45].

Adapters and low-quality sequences were trimmed from raw reads using BBMap v38.32 [47]. Trimmed reads were assembled using SPAdes v3.5.0 [48]. Attempts to annotate the MSDIN genes in genomes using existing software failed, even when gene annotators were trained on sets of known MSDINs. Therefore, we developed our own pipeline to identify our genes of interest.

### MSDIN identification pipeline

To identify MSDIN sequences in genome assemblies, we created a customizable bioinformatic pipeline. Resulting inferences of MSDIN presence/absence from assembly data were validated based on alignment data, as described below. Briefly, a set of known MSDIN sequences was used in an initial tBLASTn [49] search of genomes (Table S2). Hits with e-values below 100, a cutoff consistent with a published study [43], were translated in all reading frames and scanned for MSDIN-like motifs using MAST trained on leader and follower motifs identified with MEME [50]. Proteins where leader sequence motifs were found upstream of follower motifs and where MEME determined an e-value below 100, were retained for further analysis. All possible introns (including non-canonical GC-AG introns) were identified and resulting proteins were filtered based on known characteristics of MSDIN genes (as detailed by Walton [33]; Supplementary Methods). Our pipeline is made up of a series of self-contained and easily customizable scripts available in the Supplementary Materials. Our pipeline finds all previously identified MSDIN genes in well-studied genomes (see Supplementary Methods).

However, as a strategy to enable the identification of novel MSDINs, we chose bioinformatic pipeline parameters such that “MSDIN-like” sequences were also included in outputs. Eventually, we did exclude three MSDIN-like sequences found in the genomes of *Agrocybe cylindracea* (this species’ name is retained for consistency with NCBI but is now known as *Cyclocybe cylindracea*) and *Mycena chlorophos* from further analysis. These sequences fell at our pipeline’s minimum-cutoff value and the phylogenetic relationships to known MSDIN sequence and the lack of *POPB* in the corresponding genomes suggests they are not, in fact, MSDINs (Supplementary Results; Fig. S1).

### Validation of MSDIN identification pipeline and elucidation of locus structure

To avoid issues associated with assembly error or fragmentation, all calls of MSDIN presence/absence were validated using alignment-based methods. The files generated by our MSDIN pipeline identify MSDIN regions in target genomes, allowing us to subset alignment data. We aligned the reads from all genomes of *A. phalloides* to the reference genome using BWA MEM [51]. Read depth at MSDIN loci was determined using BEDTools [52]. For alignments without large-scale deletions, we reinserted indels and SNPs (from Wang et al. [45]) into the reference genome sequences using SAMTools faidx [53] and vcf-consensus from VCFTools [54]. Sequences resulting from the reinserting of SNPs/indels were then compared to assembly results. In instances where the presence of an MSDIN was strongly supported by alignment data but the sequence was not assembled, we inferred assembly error and inferred MSDINs directly from alignment results. When discrepancies were found between alignment and assembly results, we visualized relevant alignments alongside unaffected alignments using IGV [55]. Discrepancies between assembly and alignment data were most frequently explained by the occurrence of a heterozygous SNPs that resulted in two possible MSDIN sequences where SPAdes [48] only assembled one allele. At a small subset of loci, multiple heterozygous SNPs were not phased by variant calling software (i.e., not associated with one or the other homologous chromosome in the dikaryon). In these cases, we manually confirmed SNP phasing by visualization of all (clone-corrected) alignments, again using IGV [55].

When an MSDIN locus was not present in the reference genome, we aligned reads from all genomes to the genome with the novel MSDIN and highest quality assembly (as determined by BUSCO score). In these instances, we vetted the locus as described above. However, as these instances were rare, SNPs were not called again and instead, variants were inferred manually through visualization of alignment data in IGV [55]. In rare instances we observed misalignment of reads between very closely related loci, typically when a corresponding locus in an aligned isolate was not present in the reference.

The reinsertion of SNPs into the reference genome provided strong evidence for MSDINs cooccuring at the same locus. Locus structure was further validated by large-scale alignments of contigs (detailed in Supplementary Methods). The locus AFPHFYVPP was not assembled in the long-read reference genome but was evident in its assembly using only short reads (72 mAP). Loci were typically named based on the alleles found in the reference genome. However, the FILAPIIP locus was not found in the reference genome and its name was chosen alphabetically.

### Physical distribution of MSDINs

The physical clustering of MSDINs was assessed using a binomial expression to assess the distribution of MSDINs. Additionally, we ran a permutation test to compare the observed distances between MSDIN loci to a random distribution, as detailed in the Supplementary Methods. Correlations between physical and genetic distances were calculated with Pearson’s correlation test implemented in R. We visualized the distribution of MSDINs across *A. phalloides* individuals using the R packages ggplot2 [56], ggtree [57], and ggtreeExtra [58].

### Phylogenomics

To provide phylogenetic context to our results, we downloaded the 249 Agaricales genomes (representing 163 species) available from NCBI on November 22, 2021 (Table S3). A set of single-copy orthologs conserved across fungi (OrthoDB v9) were identified in all genomes using BUSCO [59]. We aligned BUSCO sequences using MAFFT v7.475 [60] with the setting “-auto”. Resulting alignments were trimmed using trimAl v1.2 with the “-automate1” parameter and used to construct maximum-likelihood trees with IQ-TREE v1.6.2 [61] after testing for the best-fitting model using the “-mset” parameter constrained within RAxML-compatible models. As appropriate, bootstrapping was performed in IQ-TREE using 1,000 replicates of the ultra-fast bootstrapping approximation. A consensus phylogeny of BUSCO trees was inferred with ASTRAL [62]. We used an UpSet plot generated with UpSetR [63] to compare MSDIN core sequences among species, adding data from Luo et al. [38] to identify differences and overlaps (Table S2 and S4). We established phylogenetic relationships between *A. phalloides*, *A. thiersii*, and *A. foetens* isolates used here from filtered biallelic SNP data that was thinned so that no SNPs were closer than 1 kb using VCFtools [54]. This subset of SNPs was processed using IQ-TREE as described above.

### Identification of POPB orthologs

*POPB* genes encode an enzyme required for the post-translational modification of MSDIN pro-proteins, and to identify them we obtained a set of POPB and POPA (*POPA* is a closely related gene that does not impact MSDIN maturation) protein sequences from NCBI (Fig. S2 and Table S5). We used tBLASTn to identify target regions in all Agaricales genomes, basing identification on all hits (including very short alignments) with e-values below 0.01. We extracted the 6 kb flanking either side of each hit (12 kb total; less if we ran into the end of an associated contig) using SAMtools faidx [53]. We identified candidate genes in target regions using AUGUSTUS v3.4.0 [64] with prebuilt *Laccaria bicolor* gene models and using protein cues from known POPA and POPB sequences (Table S5). All of the proteins found in target regions were again queried with known POPA and POPB sequences using BLASTp. The top 10 POPA and POPB hits (as sorted by bit score and as available, a maximum of 20 sequences) from each genome were analyzed: these protein sequences were aligned with known POPA and POPB sequences using MAFFT, trimmed using trimAl, and phylogenetic relationships among them were determined using IQ-tree, as detailed above. Resulting phylogenies were manually inspected and the presence of POPB was inferred when a candidate sequence formed a monophyletic clade with other known POPB sequences.

### Metabolite extraction

Based on preliminary bioinformatic data, three European (5mAP, 9mAP, and 29mAP) and three Californian (21mAP, 23mAP, and 75mAP) *A. phalloides* specimens were selected to represent as much MSDIN diversity as possible. A small sample of each dried mushroom was crushed to a fine powder. Each sample was extracted with 10 mL of 100% methanol and passed through filter paper. Extracts were reduced to dryness in air, weighed, and resuspended in 100% methanol at a final concentration of 1 mg/mL.

### UHPLC–HRMS and UHPLC–MS/MS analyses

UHPLC–HRMS was performed on a Thermo Scientific Vanquish UHPLC system connected to a Thermo Scientific Q Exactive Hybrid Quadrupole-Orbitrap mass spectrometer operated in positive ionization mode. A Waters Acquity UPLC BEH-C18 column (2.1 × 100 mm, 1.7 μm) was used with acetonitrile (0.1% formic acid) and water (0.1% formic acid) at a flow rate of 0.2 mL/min. A screening gradient method was implemented as follows: Starting at 10% organic for 5 min, followed by a linear increase to 90% organic over 20 min, another linear increase to 98% organic for 2 min, holding at 98% organic for 5 min, decreasing back to 10% organic for 3 min, and holding at 10% organic for the final 2 min, for a total of 37 min. Ten microliters of each sample was injected into the system for the analysis. Three well-known MSDIN toxins, α-amanitin, phalloidin, and phallacidin, were identified by comparing profiles to standards purchased from Cayman chemical (Ann Arbor, MI, USA). The absolute quantification of these three compounds was calculated relative to a standard curve (0.1–10 ppm).

### Population genetics and natural selection on MSDIN genes

Population genetic analyses used a clone-corrected dataset to identify demographic patterns. We calculated Tajima’s D and diversity statistics in 500 bp windows from alignment data using the PopGenome R package [65]. Differentiation of MSDIN gene content between Europe and California was calculated with Analysis of MOlecular VAriance (AMOVA) in GENALEX [66]. Genetic isolation between European and Californian samples was further validated using discriminant analysis of principal components (DAPC) executed in the poppr R package [67].

To measure dN/dS ratios, we aligned MSDIN nucleotide sequences using MAFFT and constructed phylogenies with IQ-TREE using parameters described above. Sequence alignments were reformatted using Pal2Nal [68]. We then estimated a single dN/dS ratio across entire phylogenies using PAML [69].

## Results

### The MSDIN pangenome within *Amanita phalloides* comprises core and accessory elements

Our MSDIN-finding bioinformatic pipeline and subsequent manual validation identified 2940 MSDIN sequences from 88 *A. phalloides* genomes (Table S1) representing 43 unique MSDIN amino acid sequences (as defined by the core region of the MSDIN that becomes the mature peptide) (Table S2). Thirteen of the unique MSDIN sequences are new (Fig. 1; Table S2). Two of these new sequences (FLPPFLP and IWGKGCDP) were found in only one individual and are not clearly associated with a specific locus; we excluded both from further analyses. Using clone-correction, we collapsed identical or nearly identical genomes into a single data point [45]. In the clone-corrected dataset we identified a total of 1308 MSDIN sequences in 38 *A. phalloides* individuals (Fig. 1). The number of unique MSDIN sequences found in these 16 European and 22 Californian individuals ranged from 25–32 with a median of 29. The *A. phalloides* MSDIN pangenome comprises 15 core-genome (found in all isolates) and 26 accessory-genome (found in only some isolates) MSDINs. Two of the accessory-genome MSDINs were found in all but one individual (Fig. 1). Assembled genomes were 94.1% complete on average but were fragmented into an average of 42,500 contigs (Table S1). The number of MSDINs found was not positively correlated with the completeness of genomes (Fig. S3).

The 41 unique MSDIN sequences mapped to 31 distinct loci. Each locus encodes one to three alleles (Fig. 1). Thirty of these loci were found in the reference genome (Table S6). We named loci based on the allele present in the reference genome assembly (see black text at bottom of Fig. 1; Supplementary Methods). Three MSDINs, IWGIGCDP (β-amanitin), AWLATCP (phalloidin), and LIQRPFAP (uncharacterized), were each found at two distinct (duplicated) loci in the reference genome. However, the genome assemblers we used typically collapse identical alleles into a single sequence. Indeed, even closely related (but different, i.e., heterozygous) alleles were sometimes only evident from SNP data. Conversely, it is possible that sequence variation near MSDINs in dikaryotic nuclei would cause two identical sequences from the same locus to appear in assemblies as two distinct alleles. While we were able to validate duplications of IWGIGCDP, AWLATCP, and LIQRPFAP in the high-quality reference genome (see Supplementary Methods), it was not possible to validate duplications of other MSDINs in other genomes. For these reasons, we chose a conservative approach and collapsed these three duplicated loci, emphasizing that the presence of more than one copy of an allele in an assembly (i.e., the “count” of a specific sequence) is suggestive of an unresolved duplication (Fig. 1).

Moreover, the loci FFPIVFSPP and FIFPPFFIPP are physically very close together and often appear to encode the same alleles, raising concerns about non-specific mapping of data. Non-specific alignment of reads from AFPHFYVPP was occasionally observed when aligning to the reference genome because in the reference genome this locus is missing (see Supplementary Methods). Similarly, a duplication of the SFFFPIP locus not present in the reference raised concerns about our ability to resolve SFFFPIP sequences (see isolates with counts of three alleles, Fig. 1). To avoid interpreting errors associated with these putatively unresolved duplications, we did not include FFPIVFSPP, FIFPPFFIPP, AFPHFYVPP, or SFFFPIP in further analyses. While our results begin to disentangle the locus structure of MSDINs, emerging long-read technologies will be necessary to fully resolve these closely related sequences.

Californian and European collections are genetically distinct, suggesting no single European site sampled here is the source of the invasive death caps we sampled in California (Fig. S4). To clarify if differences in the frequencies of accessory-genome alleles between native and invasive specimens reflect different evolutionary histories, we used AMOVA to test for significant partitioning of genetic variance at MSDIN loci. The frequency of MSDIN alleles was significantly differentiated across all loci (*p* = 0.001, Φ_PT_ = 0.31). Individual comparisons identified four significantly differentiated loci (*p* < 0.005 after multiple-comparison correction), each of which has Φ_PT_ > 0.24 (Fig. 1). The core sequences IFLVFPIPP, LPILPIPPLP, GVILIIP, GFFPPFFFPP, and FFLIVFFPP are only found in European specimens. Consistent with potential founder effects in the invasive Californian population, no MSDIN sequences are unique to California. However, some alleles are found more frequently in California, for example the IVGILGLP allele of the IIGILLPP locus is the dominant allele in California but is relatively rare among the European specimens (see bar graph at top of Fig. 1). Europe is unlikely to be a single panmictic population [21], and more collections are needed to clarify how MSDIN allele frequencies in the invasive range have changed relative to the currently unknown source population(s) within Europe.

### Both core- and accessory- genome MSDIN genes result in mature proteins

MSDIN peptides are notorious because of the extreme toxicity of a few common peptides and/or their derivatives. Lethal toxins include α-amanitin (IWGIGCNP), β-amanitin (IWGIGCDP), phalloidin (AWLATCP), and phallacidin (AWLVDCP) [33]. The genes encoding these peptides are highly conserved constituents of the pangenome (Fig. 1). LC–MS/MS profiles of six extracts clearly demonstrate that each of the corresponding mushrooms had synthesized α-amanitin, phalloidin, and phallacidin, although the relative amounts differed among the samples (Fig. S5). Whether genetic differences among mushrooms impacts variation in toxin production is unknown, but ecological and developmental variables do contribute to the amounts of toxins found in sporocarps (reviewed by Walton et al. [33]). A chemically undescribed MSDIN-product, cycloamanide G (GFFPPFFFPP), was also identified in the extracts of the 5mAP mushroom sampled in Italy (Fig. S6). This sequence matched exactly a newly identified accessory gene (Fig. 1). For simplicity and consistency with past work [33], we refer to all MSDIN compounds with common biogenesis as “cycloamanides” even though post-translational modifications sometimes place mature MSDIN products into different chemical classifications.

### Evidence for natural selection within the MSDIN gene family

**Fig. 2:**
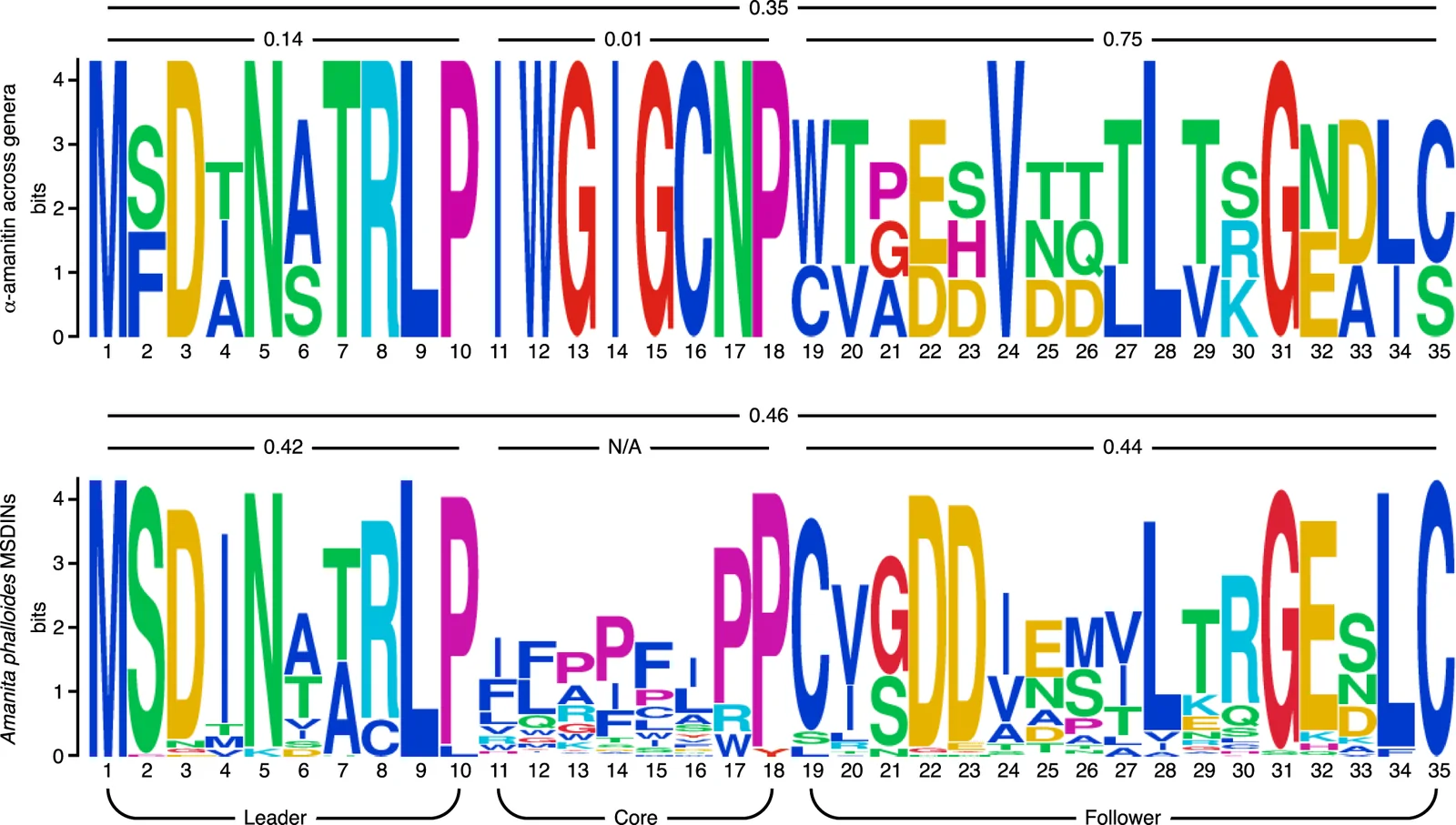
Sequence logos illustrating conservation of amino acid residues across the α-amanitin MSDIN sequences of *Lepiota venenata*, *Galerina marginata*, and *Amanita phalloides* (top) as compared to an alignment of representative sequences from the 43 unique MSDIN core sequences found within *A. phalloides* (bottom). Larger letters in the sequence logo correspond to a greater frequency of the specific amino acid residue at a given site. We inferred selection acting on leader, core, and follower portions of MSDINs and across entire sequences using dN/dS ratios (displayed above logos) as calculated in PAML. We were unable to measure selection acting on *A. phalloides* core sequences (bottom, middle) because informative sites are saturated across this highly diversified region.

Comparisons of MSDINs across genera and within *A. phalloides* reveal toxin genes are under selection. Strong purifying selection acting on the core α-amanitin sequence is evident in comparisons across genera (Fig. 2). Within *A. phalloides*, highly conserved leader and follower regions also suggest purifying selection, while highly variable core regions may reflect diversifying selection (Fig. 2). We cannot rule out neutral evolution as a driver of diversification in the MSDIN core region because its informative sites were saturated in all alignments. We did not test for selection acting on individual MSDIN core sequences within *A. phalloides* because dN/dS ratios are not reliably monotonic within species (reviewed by [70]). Selection can also be inferred from population-level metrics of the site frequency spectrum. However, our sampling strategy focused on contextualizing the diversity of a single Californian population within the breadth of European diversity. Because of the differences in sampling between California and Europe we are hesitant to interpret estimates of Tajima’s D associated with specific MSDIN loci falling within individual 500 bp windows (although we do present these values in Supplementary Results and Fig. S7). Future studies specifically designed to address patterns of selection are needed to offer robust evidence of the trends we identify at individual loci. Genome-wide patterns in the distribution of all sliding windows show a positive shift in the distribution of Tajima’s D in the Californian population. The shift may indicate the loss of rare alleles after a founder event and suggest there has not been enough time for population growth to recover rare alleles. A near-zero median of Tajima’s D from European samples is consistent with genetic equilibrium. The distribution of genome-wide Tajima’s D measurements (Fig. S7) supports the natural history of an introduced California population and a native European population, although sampling-related and demographic influences cannot be ruled out entirely (e.g., uneven sampling across unrealized population structure can result in positive shifts in Tajima’s D—an explanation we find unlikely for our geographically limited sampling in California; Fig. S7, Supplementary Results) [21].

MSDIN sequences possess very low numbers of effective codons relative to other genes in the *A. phalloides* reference genome (Fig. S8), and one hypothesis suggests selection acting on MSDIN genes has optimized MSDIN codons [71]. However, patterns in MSDIN genes do not consistently reflect genome-wide patterns of codon bias (Fig. S8) and we suggest there may be other explanations. Differential codon usage can allow for coregulation of genes [72] and can contribute to the diversity of resulting amino acid sequences by increasing mutation rates [73]. The latter function has been suggested for cyclic peptides from cone snail venoms [74]. Further work is needed to clarify the importance of codon usage in MSDIN sequences.

### MSDIN loci are physically clustered in genomes of *A. phalloides* and other *Amanita* species

**Fig. 3:**
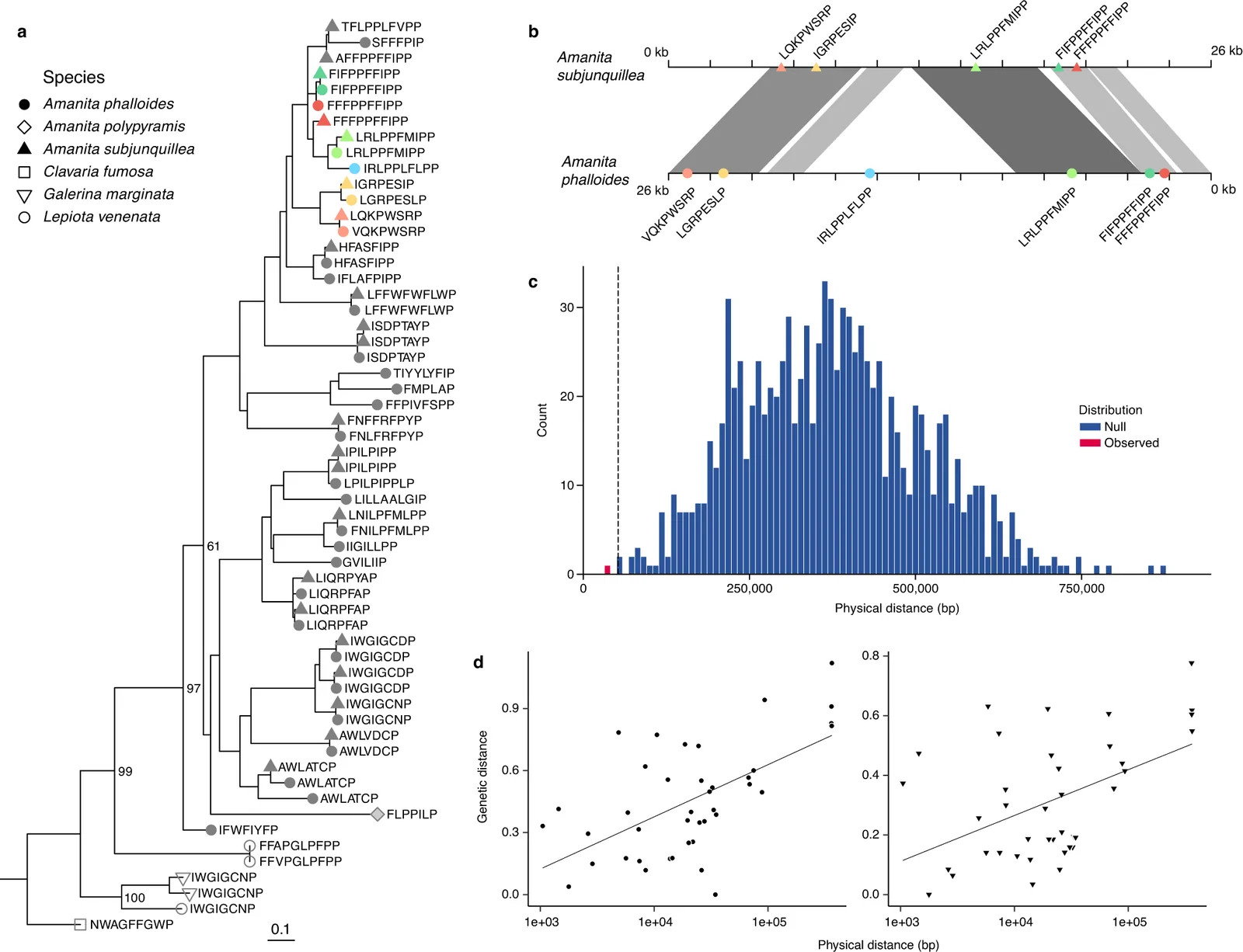
The MSDIN gene family expansion occurred independently across Agaricales genera and resulted in physically clustered loci. **a** Maximum-likelihood tree depicting the genetic relationships among MSDIN core sequences of four genera as determined from full-length sequences including the intron. The tree is rooted at the early-divergent fungus, *Clavaria fumosa*, to reflect known relationships between species. Bootstrap support values are included for a subset of nodes to emphasize that topologies within the clade containing *Amanita* spp. MSDINs are not fully resolved. Some *Amanita* spp. have been left out for readability. A full set of bootstraps values across all species’ MSDINs are presented in Fig. S9. Tips are color coded to match **b** An example of a single region encoding multiple MSDIN loci that are colocalized in both *A. phalloides* and *A. subjunquillea* genomes. **c** Permutation analysis of physical distances between MSDIN loci in *A. phalloides* indicates that the observed distance between MSDINs (red) is significantly less than a null distribution (blue) (*p* < 0.001 as indicated by the dashed line). **d** Genetic distances from the entire MSDIN-coding (left) or intron (right) sequences are significantly correlated with physical distances (for correlation test *p* < 0.001 *r* = 0.6, *p* = 0.002, *r* = 0.49, respectively; for best-fit lines *R*^2^ = 0.414 and *R*^2^ = 0.349, respectively).

To document the physical distribution of MSDINs resulting from MSDIN gene family expansions in *Amanita* spp. we first used 100 kb windows to scan the *A. phalloides* reference genome. We identified two windows containing two MSDIN loci each, three windows containing three loci each, and two windows containing six loci each (Table S6). Overall, MSDIN loci are physically clustered (binomial expression, *p* < 0.000001). We compared the observed mean distance between MSDIN loci to a randomized distribution and confirmed MSDIN loci are significantly closer together than can be explained by random chance (*p* < 0.001) (Fig. 3). Moreover, MSDIN sequences encoded at physically clustered loci are more closely related to each other than to sequences at more distant loci, i.e., we find a significant correlation between genetic and physical distances using both full coding sequences (*p* < 0.001 *r* = 0.6) and the intron sequences alone (*p* = 0.002, *r* = 0.49) (Fig. 3).

The MSDIN loci of other species are also physically clustered. In *Lepiota venenata* two novel MSDIN sequences (see below) are located within 5 kb of each other. Five contigs in *A. bisporigera* each encoded two or more MSDIN loci within 15 kb, although our ability to map MSDIN loci in this genome was hindered by extensive sequence fragmentation. In the high-quality *Amanita subjunquillea* genome, we identified four genomic regions with physically clustered loci, including one region encoding five MSDINs within ~13 kb. Alignment of the *A. subjunquillea* and *A. phalloides* assemblies revealed this region is orthologous to a ~25 kb region in the *A. phalloides* reference genome encoding six MSDIN loci (Fig. 3). In this region, the IRLPPLFLPP locus from the *A. phalloides* reference is missing in the *A. subjunquillea* genome. At least one of two alleles at this locus is present in all *A. phalloides* isolates (Fig. 1). Whether there is intraspecific variation in the presence of this locus in *A. subjunquillea*, if it has been lost from *A. subjunquillea*, or if it evolved in *A. phalloides* after the divergence of these two lineages, remain open questions.

Phylogenetic analysis of MSDIN sequences from *Amanita* spp. and *Lepiota* suggests a dynamic evolutionary history. While coding sequences may be subject to selection, MSDIN intron sequences are too short (52-58 bp) to give reliable phylogenetic resolution, so we opted to use end-to-end sequences including both coding sequences and introns. In this phylogeny, the MSDINs of different genera formed discrete groups (Figs. 3 and  S9), suggesting gene family expansions occurred independently in each genus. The clustered sequences of α-amanitin of *L. venenata* and *G. marginata* are the single exception to the general pattern.

### Taxonomic distribution of MSDINs and POPB

**Fig. 4:**
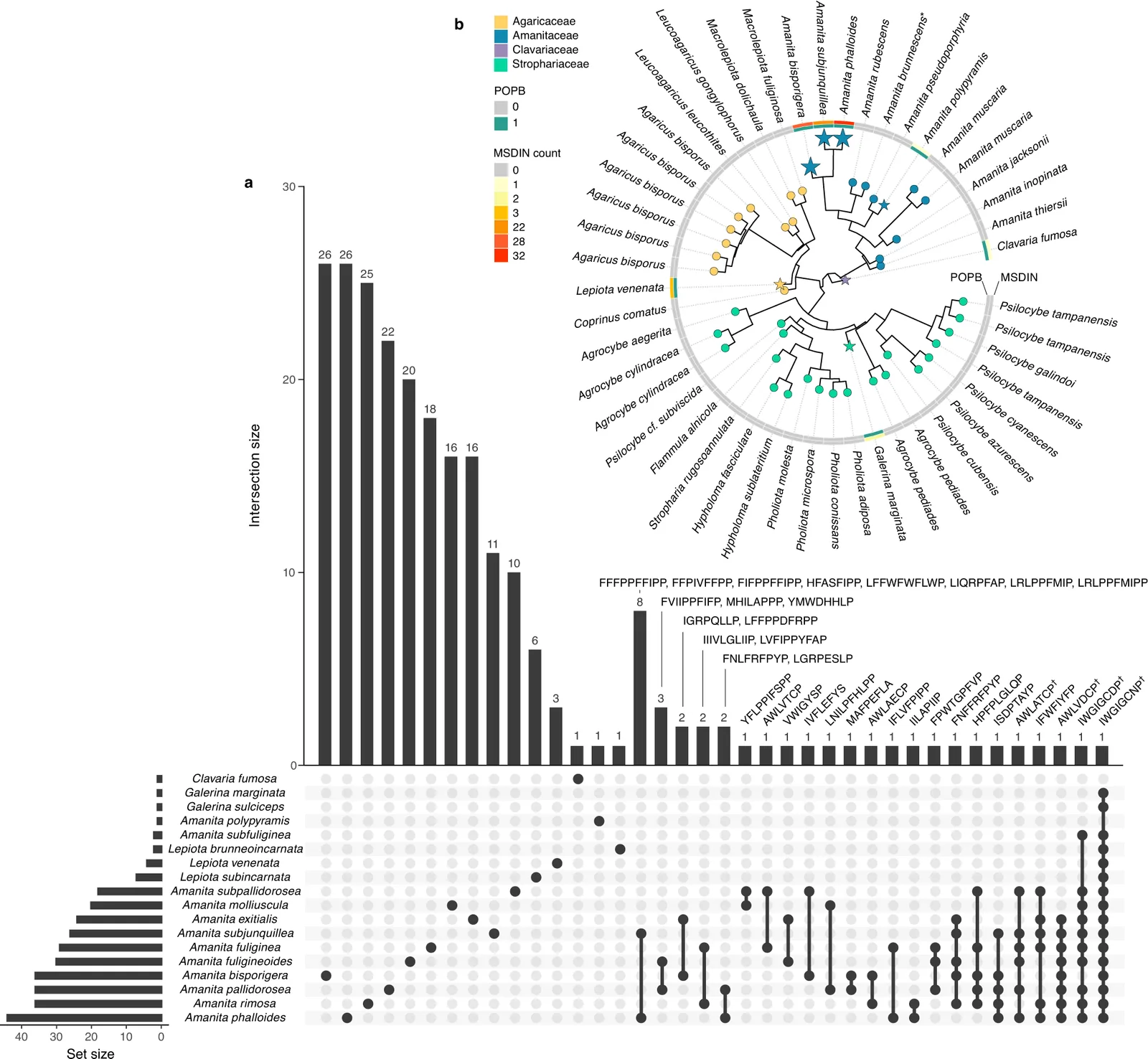
The distribution and overlap of MSDIN genes across Agaricales spp. **a** An UpSet plot depicting the overlap in MSDIN core sequences between species based on our analyses and previously published results (Tables S2 and S4). Set size reflects the total number of unique MSDIN core sequences reported for all genomes of a species; differences in this metric should be interpreted cautiously because species may differ in number of available genomes. Intersection size depicts the MSDINs present in the species marked with a solid black circle(s) underneath each column (e.g., *Amanita bisporigera* has 26 MSDINs not found in other species; the MSDIN IWGIGCNP (α-amanitin) is found in all species except *Clavaria fumosa* and *Amanita polypyramis*). MSDINs identified from published studies were not re-validated. **b** Phylogenetic relationships of MSDIN-producing species across Agaricales determined from the consensus of 289 maximum-likelihood trees of single-copy orthologs’ protein sequences (i.e., BUSCOs). Terminal branch lengths are not calculated. All nodes separating species have posterior probabilities above 0.93. The presence of the MSDIN associated processing enzyme, POPB, and counts of MSDIN sequences are depicted in the inner and outer rings (respectively). While we searched 249 genomes for MSDINs (Table S3), the tree only includes taxonomic families with species found to have at least one MSDIN and POPB. Genomes found to have both MSDIN and POPB genes are highlighted with a star, where the size of star also reflects the total MSDIN count found in each species. ^†^ These MSDIN sequences are associated with mature products with characterized bioactivity as toxins. From left to right: phalloidin (AWLATCP), phallacidin (AWLVDCP), β-amanitin (IWGIGCDP), and α-amanitin (IWGIGCNP). * The genome of *Amanita brunnescens*, a species without MSDINs, encodes a sequence found in a small and early-divergent POPB subclade (Fig. S2). More work is needed to clarify if this gene is functional.

The genomes of all known MSDIN-producing fungi encode two prolyl oligopeptidase (POP) enzymes. The first gene, *POPA*, may be widely distributed across Agaricales and is thought to function as a proteolytic housekeeping gene (a hypothesis suggested by Walton [33]). However, the prolyl oligopeptidases found among Agaricales spp. may not be orthologous [37]. The second gene, *POPB*, has previously only been found in MSDIN-producing species, where it is required for maturation of the MSDIN pro-protein. We confirmed *POPB* orthologs in all available genomes of previously identified MSDIN-producing species (Fig. 4). We also identified a *POPB* ortholog in the genome of *Amanita polypyramis* (Fig. 4), the first time a *POPB* gene has been found within the genus *Amanita* but outside of the monophyletic clade of the “lethal *Amanitas*” [33]. In addition, we discovered a *POPB* gene in *Clavaria fumosa*, an early-diverging Agaricales fungus. Phylogenetic analysis confirmed the identity of both *POPA* and *POPB* in all species where they were found (Fig. S2). Our analyses also reveal that some species have expanded sets of POP genes, including some that are closely related to *POPB* (Fig. S2), suggesting the expansion of the POP gene family as another target for future research. One small, early-diverging subclade within the larger POPB clade contains a single *Amanita brunnescens* sequence (Fig. S2). All other species with representatives in this subclade have additional POPB sequences nesting inside the larger POPB clade (e.g., *A. phalloides* has two putative POPB sequences), raising questions about the functionality of these genes. More work is needed to clarify the potential functions and evolution of *POP* genes in genomes without MSDIN sequences.

Consistent with our discovery of *POPB* genes, the *A. polypyramis* and *C. fumosa* genomes each encode a single, previously unidentified MSDIN (MSDINATRLP FLPPILP HYAPDDVNYTMLSDSLC and MFDTNDTRVP NWAGFFGWP CSPDTAGDTLNRGKDLC, respectively) (Fig. 4). We consider the identification of a second putative MSDIN in the *A. polypyramis* genome (MSNVNATRIP GPRPLAFP FFGDEENNALNCGESLC) as inconclusive because of low-quality sequence in its core and follower region. While both of these genomes appear to be missing the canonical α-amanitin gene, its apparent absence may be caused by incomplete genome assemblies.

The α-amanitin gene has been reported as the only MSDIN in *L. venenata* [40], but in his book, Walton [33] reported six MSDIN genes in *Lepiota subincarnata*. We identified two additional MSDIN sequences (MSDANNTRLP FFVPGLPFPP WTGENADHILARSKDLC & MSDANNTRLP FFAPGLPFPP WTGENADHILARSKDLC) in the published [40] *L. venenata* genome (Fig. 4), in addition to the α-amanitin gene (MDANATRLP IWGIGCNP WTPESVNDTLTKDLS). These sequences were also identified by Luo et al. [38], and by correctly parsing introns our results have resolved follower sequences. A fourth MSDIN sequence (MSDLNNTRLP VVTVLFTPPP WSGESVDHSLTRSKDLC) located within 2 kb of these sequences was only found when increasing the possible intron range parameter of our scripts by 30 bp. It is unclear if the long intron length of this MSDIN is biologically relevant or if this result reflects sequencing error, as there is a ~30 bp stretch of low-quality sequence within the corresponding sequence data. Our finding emphasizes the usefulness of our automated MSDIN-finding pipeline. It can both identify MSDINs on a large scale and recognize previously overlooked sequences.

## Discussion

The dynamic evolution of toxin genes among death caps in California and Europe suggests toxicity and the presence or absence of MSDINs are not neutral: different individuals possess different suites of genes, genes result in measurable phenotypes, and at least some sequences (including the sequence encoding the paradigmatic MSDIN α-amanitin) experience strong natural selection. Ongoing range expansions [75] emphasize a critical need to understand the ecological role of toxins produced by the fungus in native and invaded habitats. Our MSDIN-finding bioinformatic pipeline elucidates patterns not only within *A. phalloides*, but also across the *Agaricales*, creating a robust foundation for future experiments, including tests of both the “enemy-release” [76] and novel-weapons [31] hypotheses.

Cyclic peptides and structurally analogous compounds similar to the toxins found in death caps are present in the venoms of several animal species including cone snails, snakes, and spiders [33], a striking example of convergent evolution. In animals, venom diversification is often attributed to selective pressures imposed by the differential susceptibilities of diverse prey to specific toxins [77, 78], and the number of venom constituents is associated with dietary breadth [79]. Our data suggest similar dynamics for the selective pressures acting on MSDIN genes (Fig. 2). While the leader and follower regions of the *A. phalloides* MSDIN gene family are under strong purifying selection, core regions are diversifying. Core regions are so diversified that we were unable to align sequences and clarify if diversity results from neutral evolution or positive selection. While core sequences as a group are diverse, some core sequences are conserved across genera (Fig. 4a), for example α-amanitin. Strong purifying selection clearly acts on the sequence encoding α-amanitin (Fig. 2). Accessory genome constituents may be maintained by relatively rare or inconsistently distributed selective pressures, while core-genome constituents may reflect widespread selective pressures found across the death cap’s range. The allele frequencies of venom-encoding genes in snakes are thought to be adaptive in some populations, driving venom diversification through balancing selection between groups [80]. We speculate that selection on MSDINs results from interaction between specific gene products (i.e., redundancy, synergy, and density-dependence) and adaptation to local ecological conditions (e.g., fungivore populations). However, while the MSDIN core-genome comprises most of the notorious MSDIN toxins, the bioactivity of most core and accessory MSDINs remains unknown; a subset of MSDINs may have no ecological function [81]. Until the function of MSDINs in nature is defined, a holistic understanding of their evolution will remain out of reach.

The evolutionary history of MSDINs among Agaricales fungi (Figs. 3 and 4) also offers striking parallels to the evolution of knottins, a cycloamanide-like group of compounds in spider venoms. A knottin common ancestor is thought to have undergone multiple independent diversification events among spider species [82]. Similarly, the distinct clustering of MSDIN sequences from different genera (but we note the exception of *L. venenata* and *G. marginata* α-amanitin sequences) suggests MSDIN diversification occurred independently in *Lepiota* and *Amanita* (Fig. 3). Gene duplication and subsequent positive selection has been suggested for the diversification of knottins [83]. Closely related MSDIN genes are physically near each other within genomes (Fig. 3), suggesting duplication and subsequent divergence as a mechanism driving the generation of new genes in the *Amanita* as well [84]. The knottin common ancestor is hypothesized to have had a disulfide bond conferring potent bioactivity [82]. Similarly, the potency of α-amanitin is attributed to a tryptathionine linkage between cysteine and tryptophan; α-amanitin is suggested as ancestral to all known MSDIN sequences [33]. In both knottins and MSDINs, the chemical bridges are not present in all descendants of the ancestral toxins. Our results highlight the remarkable parallels between fungi and animals, illustrating common trajectories in the evolutionary histories of convergently evolved but structurally similar compounds.

Walton (2018) suggests the discontinuous distribution of MSDIN genes across *Agaricales spp*. (Fig. 4) is a result of horizontal gene transfer (HGT), as the alternative hypothesis of extensive gene loss is difficult to reconcile with the genes’ strong bioactivities. Among *Amanita spp*., MSDIN genes have only been described within the monophyletic clade of the lethal *Amanita* [85]. Our finding of both a *POPB* and an MSDIN gene in the genome of *A. polypyramis* (a species outside of the lethal *Amanita* clade) either substantially pushes back the time at which MSDINs were transferred to *Amanita*, necessitating further inference of gene loss (or incomplete lineage sorting), or suggests the occurrence of an additional HGT between *A. polypyramis* and another organism. The *A. polypyramis* MSDIN nests within the *Amanita* clade of MSDIN sequences (Fig. 3), suggesting this sequence may have evolved sometime after the lethal *Amanita* gene family expansion began. The clustering of the *A. polypyramis* MSDIN with other *Amanita* MSDINs is phylogenetically incompatible with the evolutionary history of the genus (Figs. 3 and 4), pointing to a HGT event within *Amanita*, although we emphasize the bootstrap support values for its position and the short sequences underlying the phylogeny leave open the possibility that true relationships between these MSDINs are congruent with vertical transmission. Phylogenetic relationships inferred from the longer sequence of the POPB protein are compatible with both vertical and horizontal transmission within the genus, because a transfer to *A. polypyramis* might have happened before speciation of the deadly amanita clade (Fig. S2). The phylogenetic relationships of both the MSDIN and POPB genes found in *C. fumosa* are phylogenetically compatible with our species tree, suggesting the genes may have more ancient origins than was previously thought (Fig. S2). More data are needed to clarify the questions our results raise about the evolutionary histories of the MSDIN and POP genes.

The complex life cycles, ploidy, and technical difficulties associated with growing and manipulating basidiomycetes in the laboratory have slowed the development of tools to identify basidiomycete SMs, precluding targeted experimentation. A recent survey of the fungal kingdom emphasizes Basidiomycota as a phylum harboring a unique set of understudied drug-like compounds [86]. Our MSDIN-finding bioinformatic pipeline enables drug prospecting within a previously inaccessible class of basidiomycete-specific SM and offers a roadmap for the development of similar pipelines in future.

## Supplementary Material

41396_2023_1432_MOESM1_ESM Supplemental Materials

41396_2023_1432_MOESM2_ESM Table S1

41396_2023_1432_MOESM3_ESM Table S2

41396_2023_1432_MOESM4_ESM Table S3

41396_2023_1432_MOESM5_ESM Table S4

41396_2023_1432_MOESM6_ESM Scripts and example input

## Data Availability

Sequence data for all *A. phalloides* specimens used here are being made available through Wang et al. [45]. Data for all other analyses are already public and are appropriately referenced in the text with details of specific accession numbers available in the Supplementary Materials.

## Code availability

Scripts associated with our MSDIN-finding bioinformatic pipeline are also available in the Supplementary Materials.

## Notes

**Supplementary information** The online version contains supplementary material available at https://doi.org/10.1038/s41396-023-01432-x.
