# Supplementary material for: Pangenomics of the death cap mushroom Amanita phalloides, and of Agaricales, reveals dynamic evolution of toxin genes in an invasive range
Source: ISME J. 2023 May 23;17(8):1236–46. doi: 10.1038/s41396-023-01432-x (PMC10356791; doi:10.1038/s41396-023-01432-x)
Supplement: Supplementary file 1 — Supplemental Materials [file 41396_2023_1432_MOESM1_ESM.docx]

**Supplemental Methods:**

MSDIN identification pipeline details

Our pipeline identifies MSDIN sequences from filtered BLAST+ (Camacho et al. 2009) converts them to proteins using EMBOSS (Rice et al. 2000) and BEDTools (Quinlan 2014) and identifies protein motifs in resulting sequences using MEME (Bailey et al. 2015) hits (see methods) based on previously published characteristics (Walton 2018). Specifically, we assumed that sequences begin with a start codon, leader sequences are 9-10 AA in length, core sequences are 6-10 AA, follower regions are 15-19 AA, a single intron that is 52-58 bp in length occurs in the last few codons of the follower sequence. The scripts process all possible introns encoded by both canonical and non-canonical (GC-AG) residues. We assumed that MSDIN core sequences end with a proline, a residue thought to be required for cyclization of final products (Luo et al. 2014, Pulman et al. 2016), although several studies have inferred MSDIN sequences that do not conform to this rule. Sequences identified based on these criteria that did not contain internal stop codons were then scored. To score sequences, we attributed a point for every match to known attributes of *Amanita phalloides* MSDINs: A single point was added when leader sequences ended in proline, for every residue matching CVGDDP and LC motifs at the beginning and end of the follower protein (respectively). Additionally, a single point was added if the follower length was between 15 and 19 AA (as variable length ranges above for introns and other criteria allows for slight variation around specified lengths impacting the parsing of sequences into regions). Based on preliminary results, we required at least five matches from these criteria. MSDINs that matched previously identified sequences were also selected. Only the highest-scoring interpretation of a single locus was taken even if multiple ‘passing’ interpretations were found. The above-defined MSDIN finding and scoring parameters are based on the best current evidence but are easily customizable in our pipeline’s scripts. An update to the pipeline was made after the composition of this manuscript to create ‘proline-free’ options (always-check and allow-no-proline) for users that will identify core regions that do not end in proline (unlike the proline-guided option used here). These options are much more computationally intensive and are of dubious biological relevance (see above). Use of this option did not result in the discovery of any new sequences beyond what is already reported in the literature (see validation in beginning of next paragraph) and was thus not incorporated into the main text.

To validate the parameters of our pipeline, we analyzed an *Amanita bisporigera* and an *A. phalloides* genome previously analyzed by Pulman et al. (2016). Our pipeline identified 24 of 27 *A. bisporigera* and 30 of 31 *A. phalloides* MSDIN genes. The four MSDINs that our pipeline did not identify lacked a canonical proline residue at the end of the MSDIN-core sequence and are thus unlikely to result in the mature cyclic peptides (Luo et al. 2014, Pulman et al. 2016) that were the focus of this manuscript. All MSDINs in this validation dataset were identified when using the proline-free option.

Locus structure and physical clustering of MSDIN genes:

To confirm that closely related MSDIN sequences (as determined from phylogenies of all MSDIN sequences) found in assemblies represented distinct loci and not duplicated assemblies of the same locus, we aligned entire contigs containing these regions to each other and examined resulting dot plots using DGENIES (Cabanettes and Klopp 2018). This same approach was also used to identify a small orthologous region between *Amanita phalloides* and *Amanita subjunquillea* where several MSDIN loci were present. To visualize this region we aligned small segments of the contigs using Kablammo (Wintersinger and Wasmuth 2015).

During the vetting of MSDIN locus structure we noticed that MSDINs were often physically clustered in the genome. To confirm this observation, we performed two analyses: First we identified the average number of genes found in a 100 kb window and calculated the probability that one of those genes was an MSDIN using a binomial distribution. Using a second binomial distribution, we then calculated the probability that more than one MSDIN gene would be found on the same 100 kb window. Second, we compared the average distance of all possible pairwise comparison of MSDIN gene on the same contig to a null distribution created by randomly assigning MSDIN genes to the location of any other gene (including non-MSDIN genes). We permuted the randomized distribution 999 times and identified the significance of the observed value as compared to the null distribution using the EDCF function of R (R Core Team 2021). In this analysis, fragmentation of genome assemblies will bias null distributions towards being smaller (making it less likely to identify significant clustering), as the largest possible distance between two points in the genome is decreased by a lack of end-to-end chromosomal resolution. While genome fragmentation of non-reference genome precluded similar inferences on a population scale, we did also confirm that physical linkage of MSDINs in other genomes was consistent with that observed in the reference.

UHPLC–MS/MS Identification of a novel MSDIN-derived monocyclic peptide, cycloamanide G

For the prediction of monoisotopic masses for each MS/MS fragments, Molecular Weight Calculator v.6.50 (Pacific Northwest National Laboratory) was used. Mostly b-type fragments were detected and determined for cycloamanide G. Both the gene encoding and the chemical structure of cycloamanide G (GFFPPFFFPP) were identified in this study. Consistent with MSDIN gene predictions (Figure 1), only one European isolate 5mAP showed significant feature matched with the core sequence GFFPPFFFPP.

**Supplemental Results:**

Population demographics and Selection acting on MSDIN sequences

Introduced populations often experience genetic bottlenecks, limiting genetic diversity. However, the nucleotide diversity of the Californian specimens is only slightly lower (pi = 0.001752) than the nucleotide diversity of specimens collected from across the European range (pi = 0.002163). The diversity found in California is particularly striking as sampling was concentrated within two adjacent sites at Point Reyes National Seashore. By contrast, the European collection included multiple specimens from Portugal and the United Kingdom, but also mushrooms from Sweden, the Czech republic, France, and other countries. The median value of genome-wide scans of Tajima’s D was -0.04 amongst European specimens and 0.51 amongst Californian isolates (Figure S7). A near-zero median value of Tajima’s D in Europe is consistent with genetic equilibrium. Positive values can suggest loss of rare alleles as would occur in a founder event. These patterns are consistent with what is known about the natural history of *A. phalloides*. However, we cannot rule out that these patterns are related to other demographic- and sampling-related processes (e.g., positive shift can result from uneven sampling across unrealized population structure). Larger-scale sampling efforts in the US and Europe may reveal additional underlying population structure that we were unable to detect from this sample.

Estimates of Tajima’s D corresponding to MSDIN loci often fell in the positive 0.05 tail of the Californian Tajima’s D distribution but in the negative tail of the European distribution (Figure S7). Interestingly, nearly all MSDINs with significantly negative Tajima’s D estimates were genes that encode characterized products. Only LGRPESLP was found in the negative tail of the CA Tajima’s D distribution while IWGIGCDP, AWLATCP, IWGIGCDP, IWGIGCNP, IFLAFPIPP were all found in the negative tail of the European distribution. Interestingly, all of the MSDINs except for IFLAFPIPP in the latter distribution are associated with known MSDIN toxins. The MSDIN loci LRLPPFMIPP, GVILIIP, LGRPESLP, LFFWFWFLWP, TIYYLYFIP, IFWFIYFP from CA and VQKPWSRP, LGRPESLP, FNLFRFPYP from Europe were found in the positive tail of the Tajima’s D distribution. While positive values of Tajima’s D can suggest diversifying selection, we note that some of these MSDINs did not have SNPs within the gene. We suggest that in those instances, estimates of Tajima’s D may reflect uneven sampling across unrealized finer-scale populations where negative selection is acting on each locus independently. We also cannot conclusively rule out that signal could also reflect selection acting on nearby genes. Future studies that can provide more geographic resolution may be helpful in elucidating these patterns. The positive genome-wide shift in Tajima’s D observed in California suggests the loss of rare alleles after a founder event, without enough time for population growth to recover these rare alleles. However, because the sampling strategies used in California and Europe are very different, we do not infer selection from the individual windows associated with MSDINs. Instead, we interpret Tajima’s D across the entire genome.

MSDIN-like sequences

To avoid missing new MSDIN sequences, we have intentionally set parameters of our bioinformatic pipeline to include some MSDIN-like sequences. A small subset of low-scoring MSDIN-like genes were manually inspected. We identified two MSDIN-like genes in *Agrocybe cylindracea*, and one MSDIN-like gene in *Mycena chlorophos* all scoring exactly at our bioinformatic pipeline’s cutoff value. These species do not have *POPB*. Phylogenetic analysis of these MSDIN sequences demonstrate that these sequences are more distantly related to any other MSDIN sequence than the genetic distance found between any other two known MSDIN genes (Figure S1), an observation that suggests these similarities to MSDIN genes arose through homoplasy not common ancestry.

Phylogenetic validation of MSDIN-like sequences was facilitated by our pipeline’s generation of intermediate files that contain nucleotide sequences. However, most manuscripts only report amino acid sequences and make it prohibitively laborious for us to validate if methodologies employed by those authors may introduce MSDIN-like sequences that arose through homoplasy.

Table S1. Isolates used in this study: their geographic origins and years of sampling. All isolates are *Amanita phalloides* unless otherwise indicated in the ‘population’ column. Determination of clonal groups was done previously (Wang et al. 2023) by identifying identical or nearly-identical genomes. The percentage of complete BUSCOs in the assembly of genomes is indicated. While isolate numbers are used here for facility, AmanitaBASE numbers should be used to reference isolates as they are consistent between studies from Dr. Anne Pringle’s lab. A single mushroom with a very poor-quality assembly (7mAP; Table S1), was used in initial scans of MSDIN genes, but was removed from subsequent analyses. [See attached tabular data file]

Table S2. Amino acid sequences of all MSDIN sequences used in this study, including those previously identified and those identified here. The presence of the prolylproline oligopeptidase gene *POPA* (thought to be a housekeeping gene) and *POPB* (required for MSDIN processing) is also indicated. The designation of a ‘new’ MSDIN reflects the core sequence. However, because sequences were filtered to be unique across the entire MSDIN (leader, core, and follower) within species, a single ‘new’ core sequence may be included multiple times if variation was found in the leader and or follower region. For example, the leader sequence of LRLPPFMMPP in *A. phalloides* had two variants leading to two entries representing this one new core sequence. Note introns do not appear to have been properly parsed in Hallen et al. 2007. [See attached tabular data file]

Table S3. Data associated with 249 Agaricales genomes representing 163 species downloaded from NCBI. Accession numbers are presented alongside genome statistics and associated taxonomic data. Taxonomy data indicated in comma-delimited format with fields indicating kingdom, phylum, class, order, family, genus, species, and isolate information when available.

Table S4. Amino acid sequences of all MSDINs identified by Luo et al. (2022). Sequences were filtered to only represent unique combination of species and core sequence that were not already represented in Table S2. Because these data were published after the composition of this manuscript, they were only added to analyses presented in the UpSet plot of Figure 4. [See attached tabular data file]

Table S5: Protein accession numbers of POPA and POPB sequences from NCBI used to train AUGUSTUS to find these genes in genomic sequences.

| **Accession** | **Protein** | **Species** |
| --- | --- | --- |
| ADN19204.1 | POPA | *Amanita bisporigera* |
| AEX26937.2 | POPA | *Galerina marginata* |
| QIM40764.1 | POPA | *Amanita exitialis* |
| QIM40768.1 | POPA | *Amanita pallidorosea* |
| QIM40766.1 | POPA | *Amanita molliuscula* |
| QIM40769.1 | POPA | *Amanita rimosa* |
| QIM40765.1 | POPA | *Amanita fuliginea* |
| QKM76206.1 | POPA | *Amanita subpallidorosea* |
| KAF8351067.1 | POPA | *Amanita rubescens* |
| KIL67398.1 | POPA | *Amanita muscaria* |
| PFH50576.1 | POPA | *Amanita thiersii* |
| AEX26938.2 | POPB | *Galerina marginata* |
| AVX48321.1 | POPB | *Amanita exitialis* |
| E2JFG2.1 | POPB | *Amanita bisporigera* |
| QKM76210.1 | POPB | *Amanita subpallidorosea* |
| QIM40772.1 | POPB | *Amanita molliuscula* |
| QIM40773.1 | POPB | *Amanita pallidorosea* |
| QIM40771.1 | POPB | *Amanita fuliginea* |
| QIM40774.1 | POPB | *Amanita rimosa* |
| QGP74450.1 | POPB | *Lepiota venenata* |

Table S6: The locations of MSDIN loci in the *Amanita phalloides* reference genome used in this study. The locus AFPHFYVPP was not assembled in the long-read reference genome but was evident in an assembly using only short reads (72mAP; see Supplemental Methods).

| Contig | Start | End | Core |
| --- | --- | --- | --- |
| Contig125_length_18784 | 13143 | 13300 | AWLATCP1 |
| Contig126_length_159408 | 108990 | 109153 | FNLFRFPYP |
| Contig13_length_320321 | 108671 | 108833 | LIQRPFAP1 |
| Contig15_length_729504 | 192570 | 192739 | LFFWFWFLWP |
| Contig15_length_729504 | 198140 | 198304 | SFFFPIP |
| Contig17_length_361105 | 21638 | 21801 | VQKPWSRP |
| Contig17_length_361105 | 23390 | 23548 | LGRPESLP |
| Contig17_length_361105 | 30431 | 30601 | IRLPPLFLPP |
| Contig17_length_361105 | 40063 | 40236 | LRLPPFMIPP |
| Contig17_length_361105 | 43830 | 44002 | FIFPPFFIPP |
| Contig17_length_361105 | 45013 | 45185 | FFFPPFFIPP |
| Contig24_length_239456 | 105439 | 105603 | FNILPFMLPP |
| Contig24_length_239456 | 109351 | 109508 | GVILIIP |
| Contig24_length_239456 | 110311 | 110477 | LPILPIPPLP |
| Contig24_length_239456 | 155296 | 155453 | AWLATCP2 |
| Contig24_length_239456 | 93004 | 93164 | LILLAALGIP |
| Contig24_length_239456 | 96249 | 96404 | IIGILLPP |
| Contig26_length_358228 | 40286 | 40451 | FFPIVFSPP |
| Contig2_length_1000849 | 729092 | 729252 | AWLVDCP |
| Contig4_length_763032 | 448147 | 448310 | ISDPTAYP |
| Contig52_length_148998 | 112278 | 112441 | IWGIGCNP |
| Contig52_length_148998 | 117889 | 118050 | IWGIGCDP1 |
| Contig52_length_148998 | 94899 | 95057 | IWGIGCDP2 |
| Contig57_length_298947 | 287318 | 287475 | FMPLAP |
| Contig57_length_298947 | 288014 | 288179 | TIYYLYFIP |
| Contig57_length_298947 | 45313 | 45479 | IFLAFPIPP |
| Contig57_length_298947 | 47226 | 47387 | HFASFIPP |
| Contig8_length_730935 | 509698 | 509860 | LIQRPFAP2 |
| Contig98_length_93664 | 74398 | 74565 | IFWFIYFP |


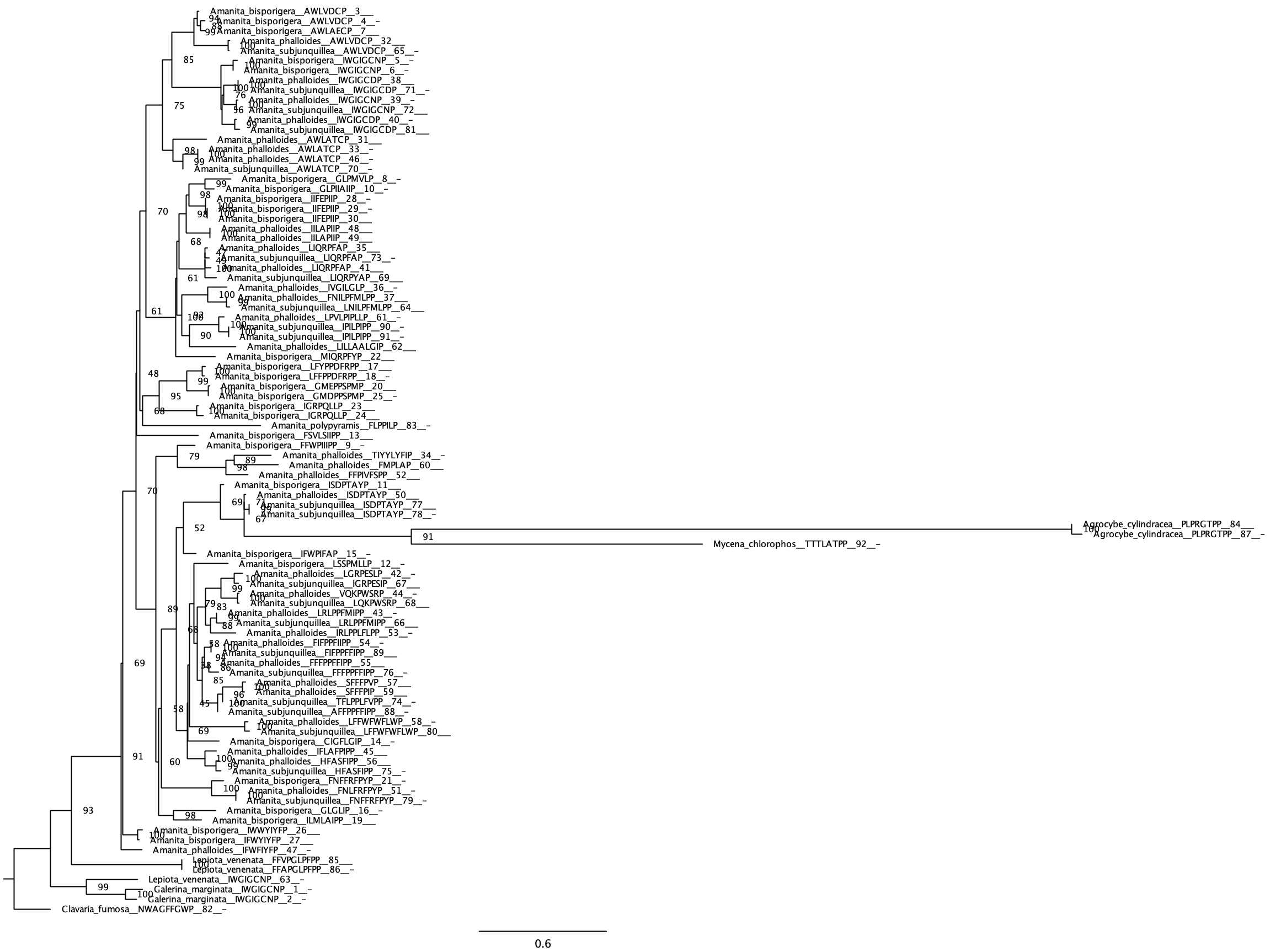


Figure S1. Maximum likelihood phylogeny of MSDIN nucleotide sequences, including full coding region and intron, comparing MSDIN sequences from Agaricales genomes used in this study. Given phylogenetic relationships of MSDIN-like sequences in *Mycena chlorophos* and *Agrocybe cylindracea* and the lack of the POPB MSDIN-processing enzyme that is thought to be required for maturation of MSDIN products, we assume that these sequences are the result of homoplasy instead of true MSDIN genes (see Supplemental methods). The phylogenies’ leaves indicate the species name, inferred MSDIN core sequence, and an arbitrary number used to count MSDIN sequences for technical reasons. Tree is rooted at *Clavaria fumosa* to reflect species relationships.


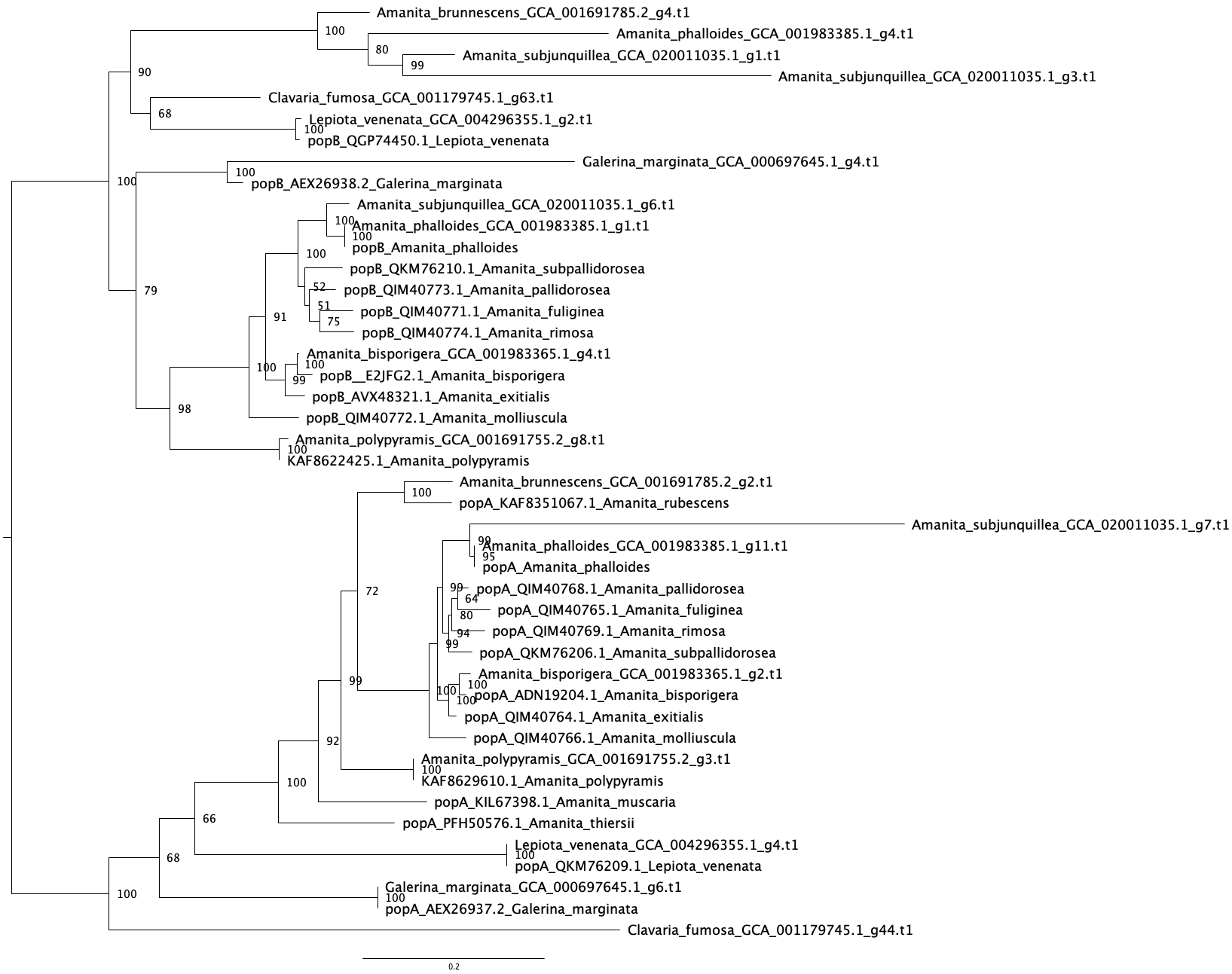
 Figure S2. Maximum likelihood phylogeny of the prolylproline oligopeptidases POPA and POPB, amino acid sequences from the genomes of Agaricales species. POPA is widely distributed across fungi and is thought to have a ‘housekeeping’ functionality while POPB is required for maturation of MSDIN-gene products. Here we demonstrate that *Amanita polypyramis* and *Clavaria fumosa* have both *POPA* and *POPB* genes. When available, previously inferred POP proteins are included for context and are denoted with the inferred POP gene, NCBI accession, and species designation. Our prediction pipeline resulted in identical, or nearly identical, sequences to past inferences (our predictions are denoted with species name, NCBI genome accession, and an arbitrary gene designation “g#”). For example, see *A. polypyramis* g3 and KAF8629610 at the top of the POPA clade and g8 and KAF8622425.1 at the bottom of the POPB clade. An additional clade of putative prolylproline oligopeptidases that branches off of the POPB sequences of *Lepiota venenata* and *Clavaria fumosa* (top of figure) appears to reflect some expansion of this gene family, at least in *Amanita subjunquillea* and *Amanita phalloides*. It is unclear what the significance the *Amanita brunnescens* POP gene in the aforementioned clade has, as we did not identify any MSDIN sequences in this genome. Further work is needed to resolve the expansion of this gene family. Two *A. phalloides* sequences were obtained from the supplement of Pulman et al. (2018) and thus do not have NCBI accession numbers.


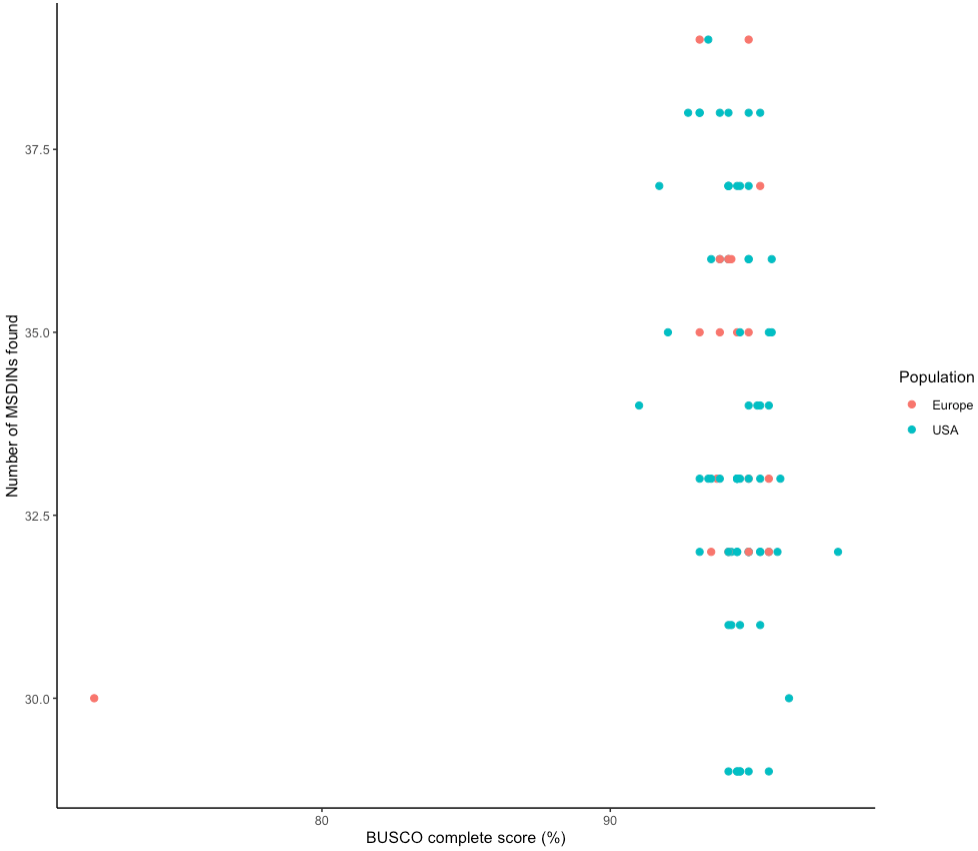


Figure S3. Comparison of genome completeness (as measured by the percentage of complete BUSCOs found) compared to the number of MSDINs that were inferred in each genome. A single genome with a BUSCO score of 10% was not included in this or any other analysis in the study. Axes have been restricted to allow for visualization of spread. Pearson’s correlation test found no significant association between variables for the full dataset ( *p* = 0.752; r = 0.034 ) or for the European population ( *p* = 0.078; r = 0.413 ). We assume that a negative significant correlation in the USA sample ( *p* = 0.015; r = -0.295 ) is spurious given that it is difficult to explain a negative association between genome completeness and the number of MSDINs found and because this pattern is not visually discernable in the plot.


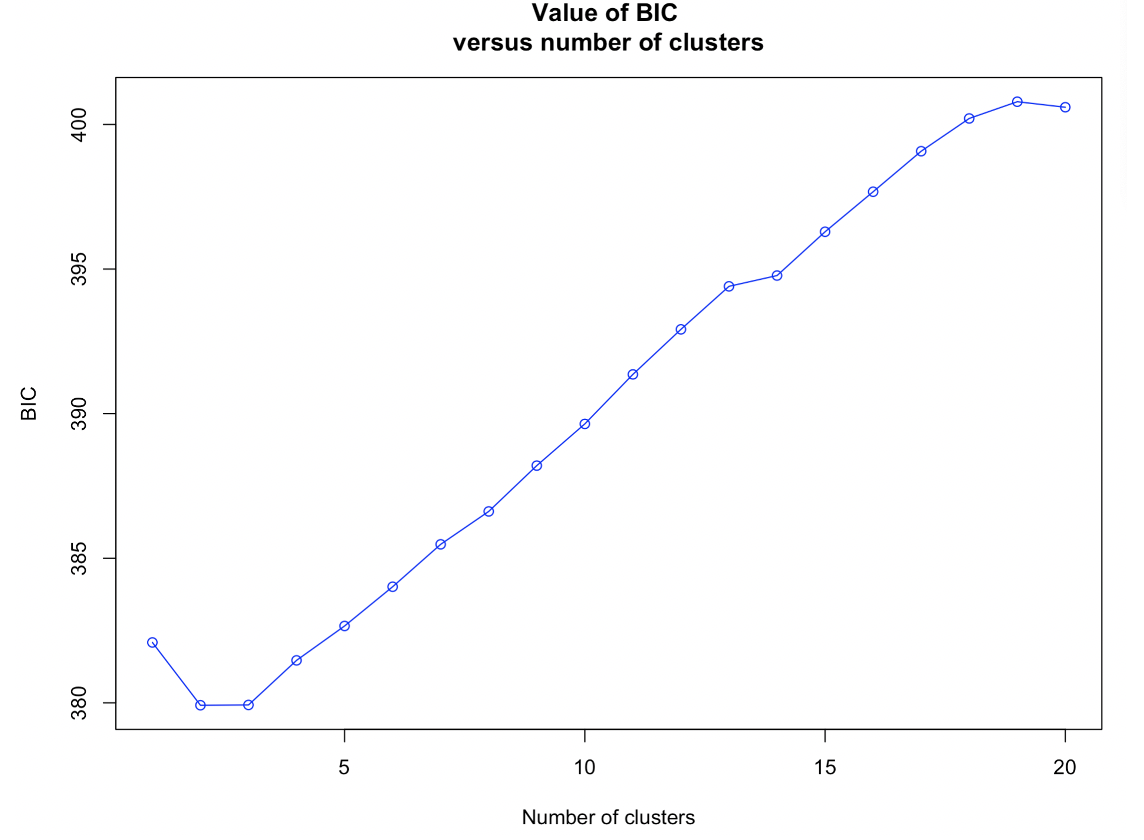


Figure S4. Plot of Bayesian information criterion (BIC) versus the number of possible clusters representing SNP data from 88 isolates of *Amanita phalloides* sampled in the California and Europe. BIC is a goodness of fit measure, whereas clusters represent potential populations. The lowest point indicates the optimal number of populations. We inferred two populations of *A. phalloides*. The third possible population separated the specimens collected in Portugal from the rest of the European sample. However, as this partition was not as strongly supported as the European/Californian split, and because our sampling scheme was not designed to clarify fine-scale population structure, we inferred two populations.


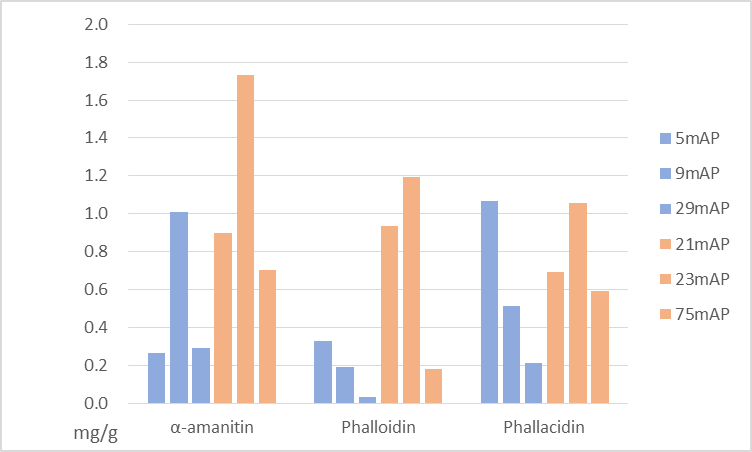


Figure S5. Absolute quantification of α-amanitin, phalloidin, and phallacidin from crude extracts of three European (blue) and three Californian (orange) mushroom specimens. Specimens appear left to right in the same order that they are indicated top to bottom in the legend.


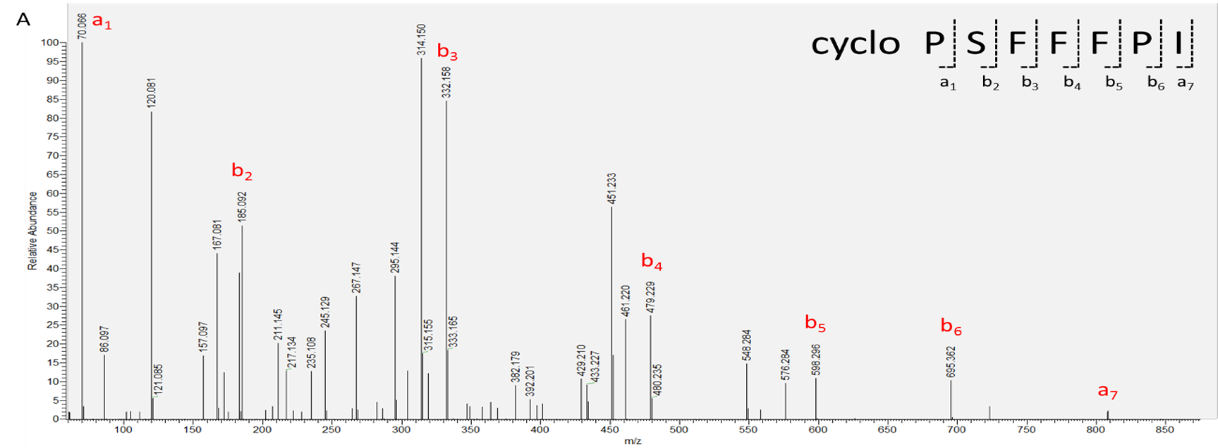

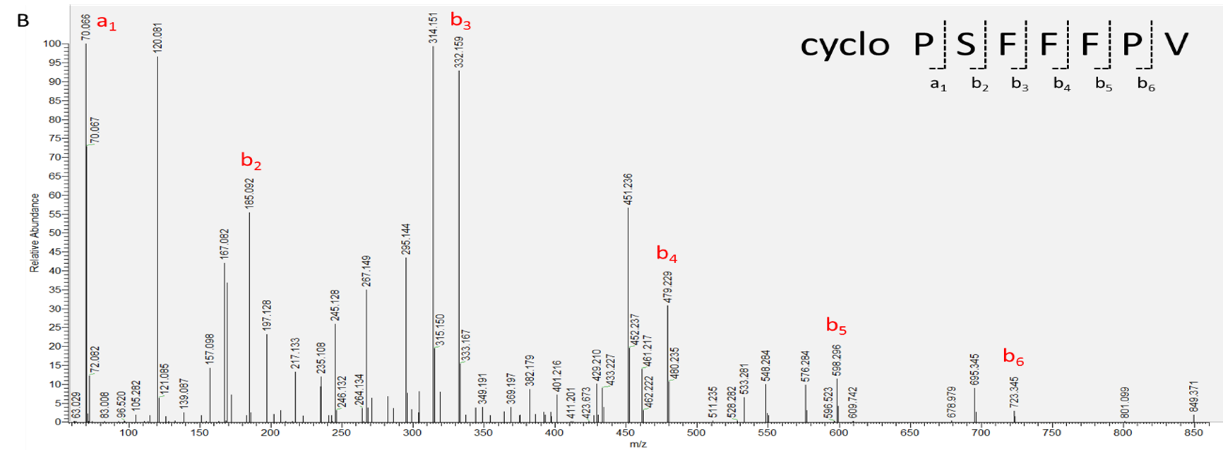

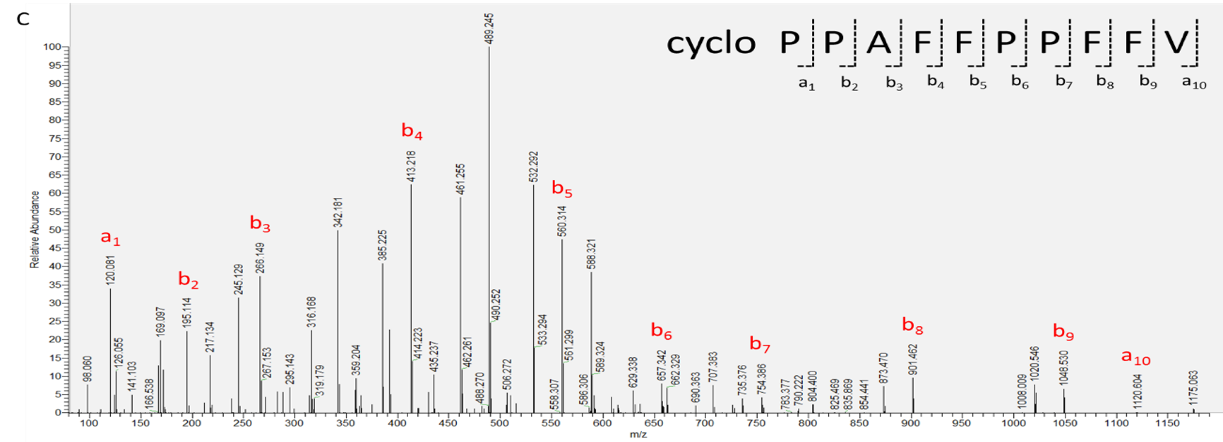

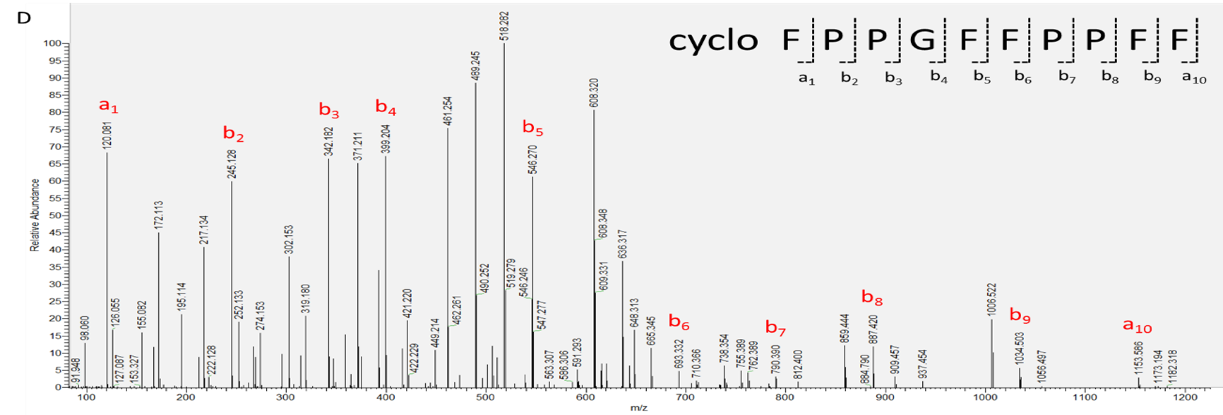


Figure S6. MS/MS fragmentation analysis of monocyclic peptides produced in *Amanita* *phalloides*. A: MS/MS fragmentation profile of cycloamanide B (SFFFPIP), B: MS/MS fragmentation profile of cycloamanide E (SFFFPVP), C: MS/MS fragmentation profile of antamanide (AFFPPFFVPP), D: MS/MS fragmentation profile of cycloamanide G (GFFPPFFFPP), a new MSDIN not reported before.


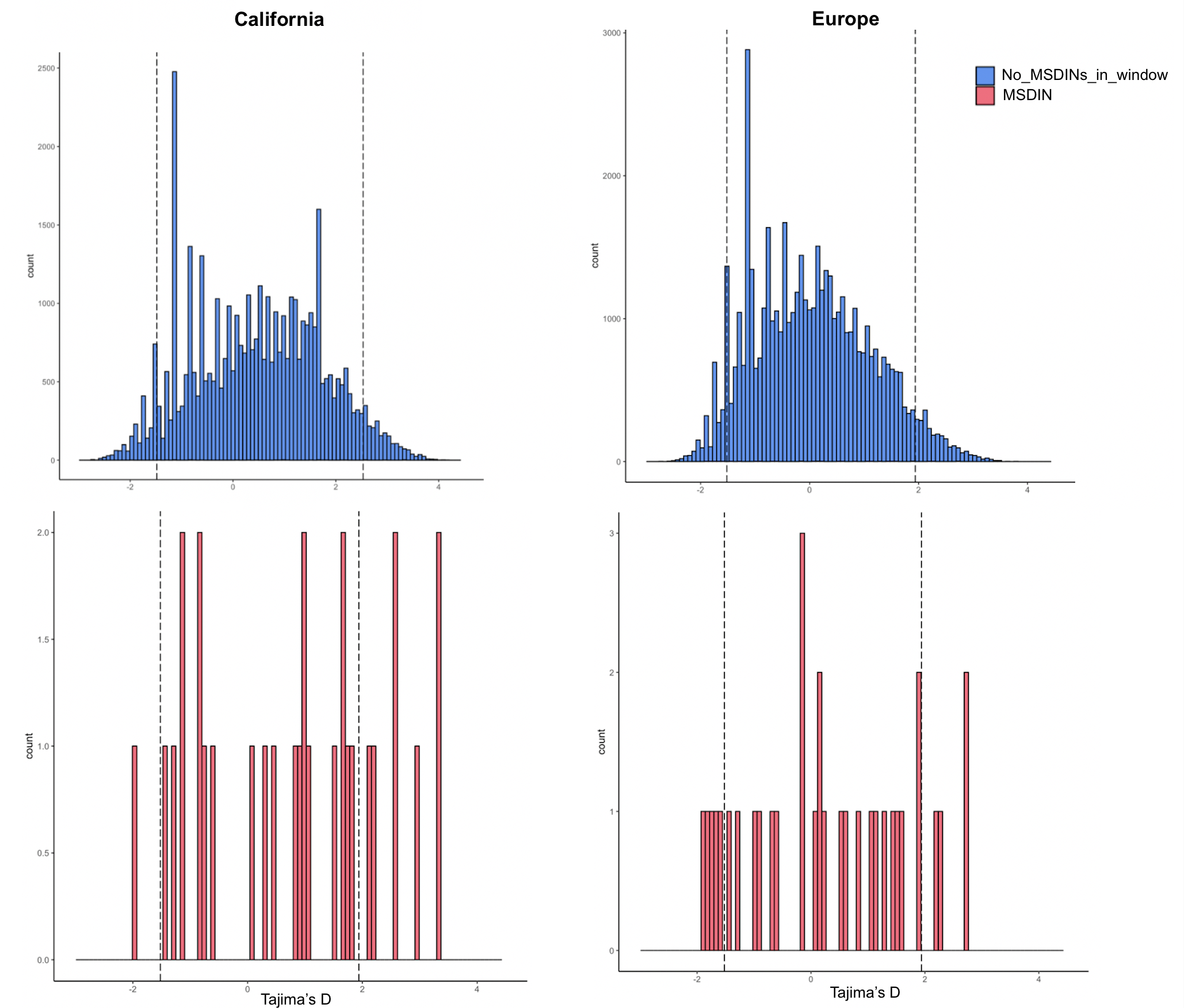
Figure S7: Distribution of Tajima’s D calculated in 500 bp sliding windows across the *Amanita phalloides* reference genomes. Values on the left correspond to a sample of isolates from California while samples on the right are European. A histogram of all values (top) is compared to windows where windows corresponded with MSDIN sequences (below). Vertical dotted lines delineate the 0.05 positive and negative tails of the entire distribution. Specific MSDIN sequences falling in these tails are detailed in the Supplemental Results


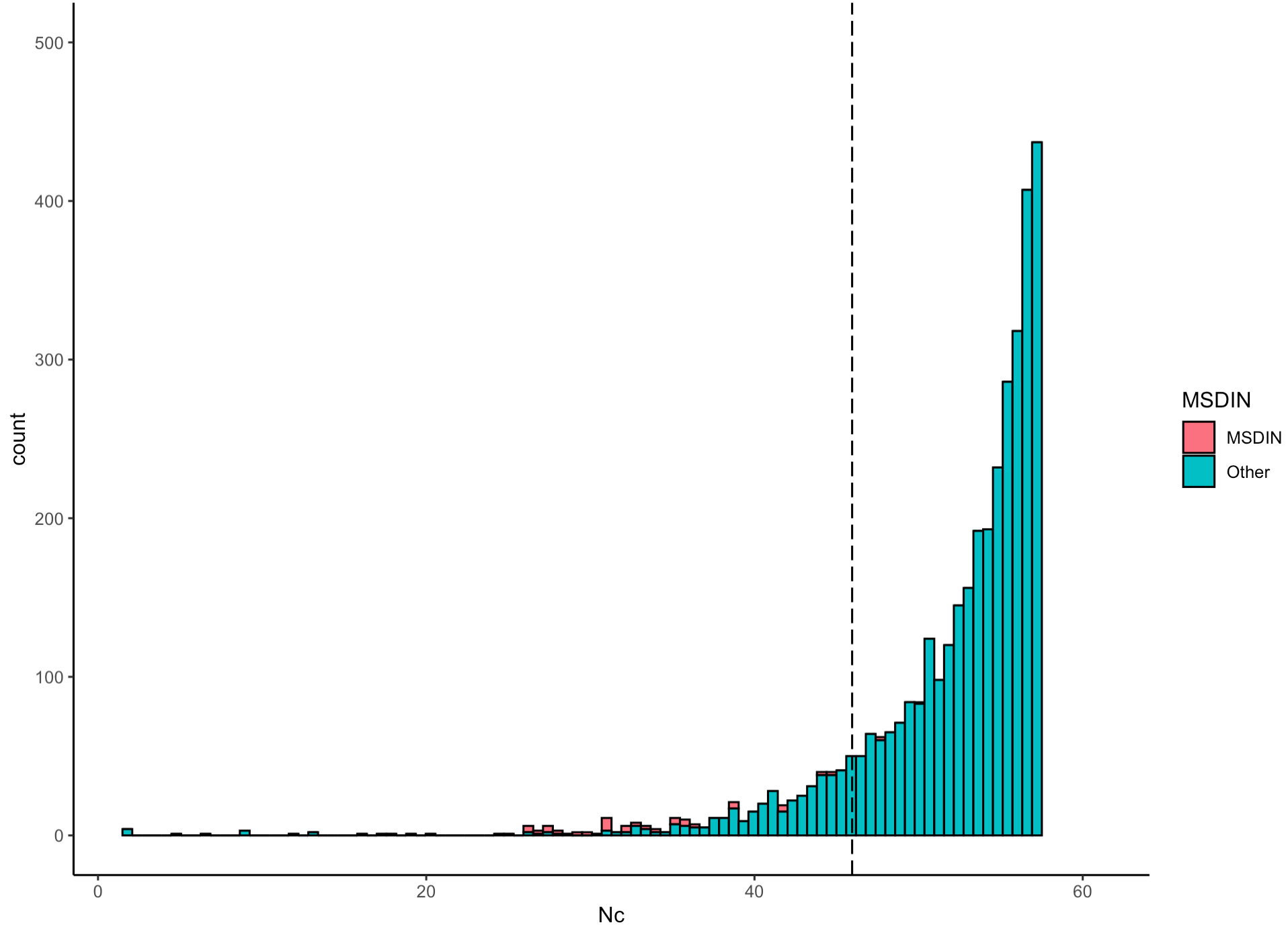

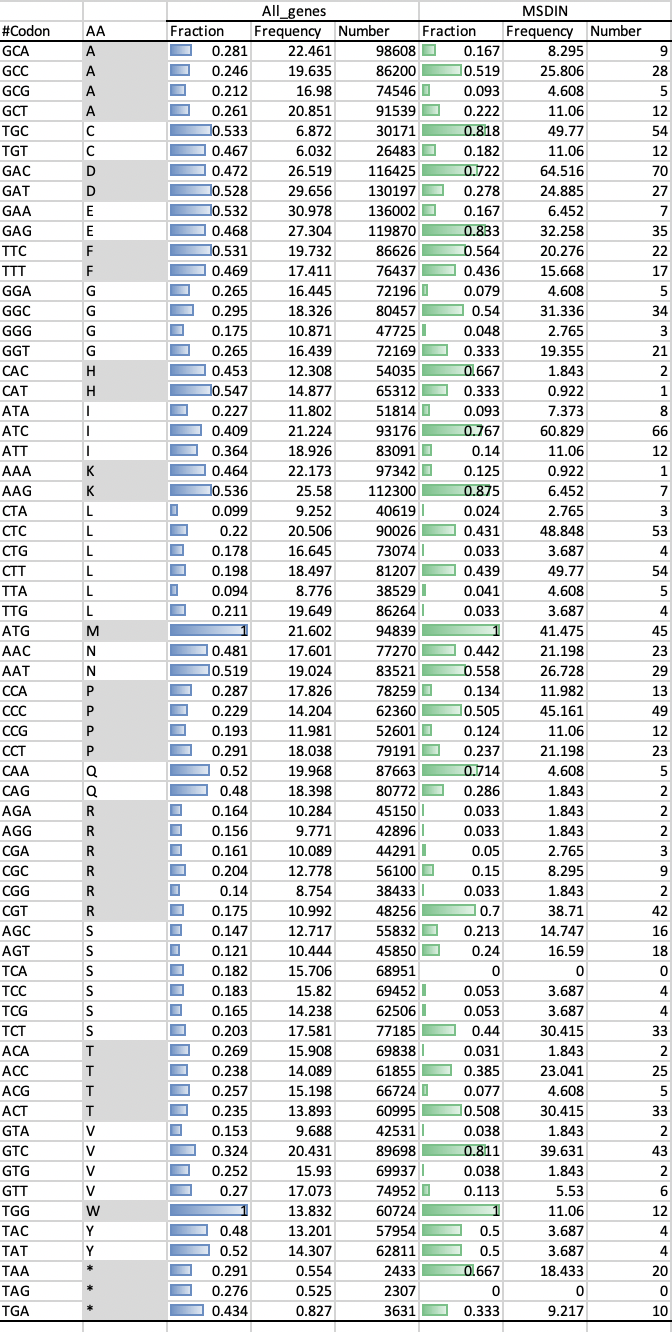


Figure S8: Codon bias in MSDIN coding sequence does not clearly reflect patterns of overall codon usage in the genome. MSDIN sequences often have low effective number of codons (Nc) (left), a pattern that can result from codon optimization. However, patterns of codon usage in MSDIN genes do not clearly align with genome-wide patterns (right).


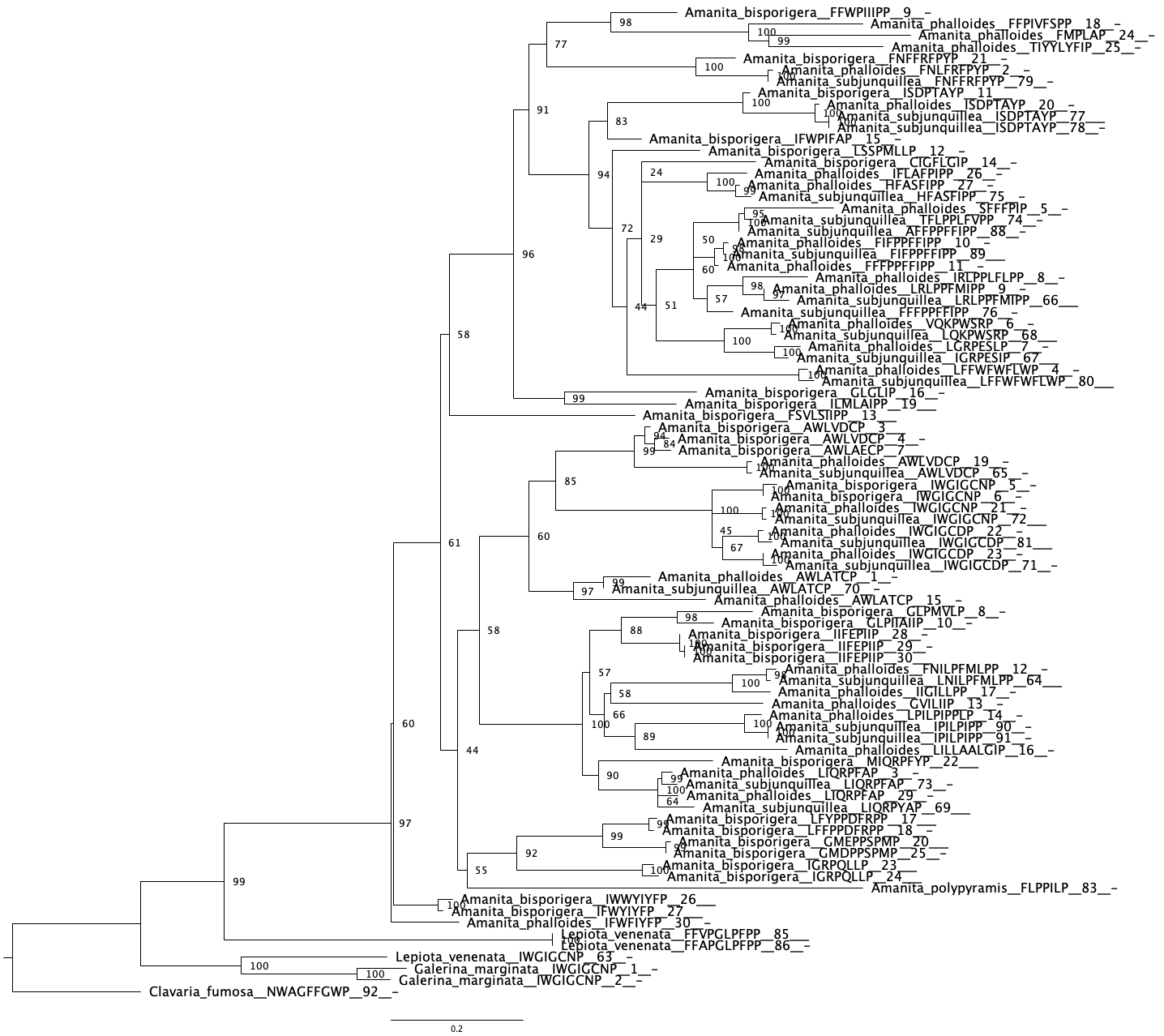
 Figure S9. Maximum likelihood phylogeny of MSDIN nucleotide sequences, including full coding region and intron from all publicly available Agaricales genomes where the *POPB* was detected. The phylogenies’ leaves indicate the species name, inferred MSDIN core sequence, and an arbitrary number used to count MSDIN sequences for technical reasons. Tree is rooted at the MSDIN found in the early-divergent fungus *Clavaria fumosa* to reflect known phylogenetic relationships of species. However, we note that there is still debate on if the MSDIN gene family’s history is concordant with species histories.

Cited Sources:

Bailey, T. L., J. Johnson, C. E. Grant, and W. S. Noble. 2015. The MEME Suite. Nucleic Acids Research 43:W39–W49.

Cabanettes, F., and C. Klopp. 2018. D-GENIES: Dot plot large genomes in an interactive, efficient and simple way. PeerJ 2018.

Camacho, C., G. Coulouris, V. Avagyan, N. Ma, J. Papadopoulos, K. Bealer, and T. L. Madden. 2009. BLAST+: Architecture and applications. BMC Bioinformatics 10:1–9.

Luo, H., H. E. Hallen-Adams, Y. Lüli, R. M. Sgambelluri, X. Li, M. Smith, Z. L. Yang, and F. M. Martin. 2022. Genes and evolutionary fates of the amanitin biosynthesis pathway in poisonous mushrooms. Proceedings of the National Academy of Sciences 119.

Luo, H., S. Y. Hong, R. M. Sgambelluri, E. Angelos, X. Li, and J. D. Walton. 2014. Peptide macrocyclization catalyzed by a prolyl oligopeptidase involved in α-amanitin biosynthesis. Chemistry and Biology 21:1610–1617.

Pulman, J. A., K. L. Childs, R. M. Sgambelluri, and J. D. Walton. 2016. Expansion and diversification of the msdin family of cyclic peptide genes in the poisonous agarics Amanita phalloides and A. bisporigera. BMC Genomics 17.

Quinlan, A. R. 2014. BEDTools: The swiss-army tool for genome feature analysis. Current Protocols in Bioinformatics 2014:11.12.1-11.12.34.

R Core Team. 2021. A Language and Environment for Statistical Computing.

Rice, P., I. Longden, and A. Bleasby. 2000. EMBOSS: The European molecular biology open software suite. Trends in genetics 16:276–277.

Walton, J. 2018. The Cyclic Peptide toxins of Amanita and Other Poisonous Mushrooms. Springer.

Wang, Y.-W., M. C. McKeon, H. Elmore, J. Hess, H. Gage, S. C. Gonçalves, W. Mao, J. Golan, L. Harrow, C. M. Hull, and A. Pringle. 2023. Invasive Californian death caps develop mushrooms and sporulate unisexually and bisexually. bioRxiv 2023–01.

Wintersinger, J. A., and J. D. Wasmuth. 2015. Kablammo: An interactive, web-based BLAST results visualizer. Bioinformatics 31:1305–1306.
