# Supplementary material for: Pangenomics of the death cap mushroom Amanita phalloides, and of Agaricales, reveals dynamic evolution of toxin genes in an invasive range
Source: ISME J. 2023 May 23;17(8):1236–46. doi: 10.1038/s41396-023-01432-x (PMC10356791; doi:10.1038/s41396-023-01432-x)
Supplement: Supplementary file 6 — Scripts and example input [file 41396_2023_1432_MOESM6_ESM.gz › ./example_input/Msdin_pipeline_scripts.docx]

The following scripts were developed by Milton T. Drott for the manuscript entitled “Pangenomics of the death cap mushroom *Amanita phalloides*, and of Agaricales, reveals dynamic evolution of toxin genes in an invasive range”. At the beginning of each script are instructions for what format programs need to be installed and what files must be in place. Please carefully read these sections and define highlighted variables as appropriate. In general, default variables should function well with previous scripts if you have not moved or renamed things. An example of library/motif files used in the manuscript as well as example output files from each step of the pipeline are provided in ‘example_input.tar.gz’.

| MSDIN_blast_new.sh |
| --- |
| #!/bin/bash  #########################################################################################  #FUNCTION  #this script will take a genome and find target regions using a set of MSDIN protein sequence it will then translate the target regions  #into all possible reading frames for processing by MSDIN_motif.sh  #  #REQUIRED PROGRAMS  #this script assumes you: have blast, bedtools, and EMBOSS installed and in PATH  #  #REQUIRED FILES  #this script requires: a single-column list of input names called “name_list” and a fasta file of query MSDIN proteins to use as queries called #“MSDIN.faa” note that these are present in the example input  #  #REQUIRED FORMATING OF INPUT  #genomes should be named “input_name_from_list”.fna.gz (gzipped) and located in the folder path noted above (defined below)  #  #  #MISC  #Please also set variables “Num_threads_for_blast” and “Genome_folder_path” at top of script.  #testing has been one using ncbi accession numbers as names results from other naming schemes may vary  #  #originally developed in OSX environment and tested on linux – see README.  #  #script will self-tidy and while I’ve tried to avoid this, you shouldn’t store any important files in the run directory as they could be remove. All #files that need to be brought in need their paths specified at the top of the script  #  #script outputs a set of proteins that may contain fasta sequences in a folder called ‘putative_msdin_prots’ that it creates  #  #The script renames chromosomes to purge naming schemes that may break the pipeline. To aid in downstream usages, the script will also create a key of #how chromosomes were renamed. This can be useful for later steps if wanting to extract sequences or refer references #in the literature that use the #original naming. Renamed genome files are currently not retained by the script but could be if you remove "$iso".fna from the rm command at the bottom #of the script.  #  ###########################################################################################  #  #Variables that may need defining ----------------------------------------------------------------  Num_threads_for_blast="16"  Genome_folder_path="./genomes/"  ReadME="NO"  #------------------------------------------------------------------------------------------------  #  if [ $ReadME != "YES" ];then  echo Please read README before proceeding  exit  fi  #  #  mkdir putative_msdin_prots  #loop over isos  while read -r iso;do  # Current_time=`date +"%T"`  echo ===============================================  echo Beginning msdin_blast of $iso at $Current_time  echo ===============================================  #  #bring in and unzip genome  cp "$Genome_folder_path""$iso".fna.gz .  gunzip "$iso".fna.gz  #  #deal with the fact that later parts of the program break if you give it a period in name – other characters may also need purging depending on naming  Working_iso=`echo $iso \| sed 's/\./_/g'`  #  #rename chromosomes and add chrm length to them…  #first rename file to have the header iso_chrm#  cat "$iso".fna \| awk -v iso="$Working_iso" -v chrm="0" '{if ($0 ~ ">") {print ">"iso"_chrm"++chrm} else {print}}' >temp.fna  #then create a file of chrm lengths to use in renaming where length is added to name  cat temp.fna \| awk '$0 ~ ">" {if (NR > 1) {print c;} c=0;printf substr($0,2,100) "\t"; } $0 !~ ">" {c+=length($0);} END { print c; }' \| awk '{print ">"$1,">"$1"_""length_"$2}' > temp_chrm_lengths  #  #add lengths to name  awk 'FNR==NR{a[$1]=$2;next} {if ($1 in a){$1=a[$1]}; print $0}' temp_chrm_lengths temp.fna > genome_with_lengths  #  #create key of chrm names – this can be useful later if wanting to extract sequences or refer back to NCBI names  grep '>' "$iso".fna > OG_chrm_names  awk '{print $2}' temp_chrm_lengths > new_chrm_names  paste OG_chrm_names new_chrm_names > "$iso"_chrm_name_key  rm OG_chrm_names new_chrm_names  #  mv genome_with_lengths "$iso".fna  rm temp.fna temp_chrm_lengths  #  #prepare blast  makeblastdb -dbtype nucl -in "$iso".fna  #  #run blast removing evalues below above 100  tblastn -evalue 100 -query MSDIN.faa -db "$iso".fna -num_threads "$Num_threads_for_blast" -outfmt "6 qseqid sseqid pident length mismatch gapopen qstart qend sstart send evalue bitscore" > blast_result  #  #go 30bp upstream of blast hit and 300bp downstream of that to grab out full region where MSDIN may be  #process resulting region into a bed file  cat blast_result\| awk -F'\t' '{if ($9<$10) {print $0"\t""+"} else {print $0"\t""-"}}' \| awk -F'\t' '{if ($13=="+") {print $0"\t"$9-30"\t"$9-30+300} else if ($13=="-") {print $0"\t"$9+30-300"\t"$9+30}}' \| awk -F'\t' '{print $1"\t"$2"\t"$14"\t"$15"\t"$13}' \| awk '{print $2"\t"$3"\t"$4"\t"$1"__"NR"\t""NA""\t"$5}' > temp_blast.bed  #  #get chrm lengths  awk '{print $1}' temp_blast.bed \| sed 's/.*length_//' \| sed 's/_cov.*//' > chrm_lengths  #  #the sort makes it so that a certain msdin start location Is only used once no matter how many hits are there.  paste temp_blast.bed chrm_lengths \| awk -F'\t' '{print $1"\t"$2"\t"$3"\t"$4"\t"$7"\t"$6}' \| awk -F'\t' '{if ($2<0) {$2="1"} {print $1"\t"$2"\t"$3"\t"$4"\t"$5"\t"$6}}' \| awk -F'\t' '{if ($3>$5) {$3=$5} {print $1"\t"$2"\t"$3"\t"$4"\t"$5"\t"$6}}' \| sort -u -k1,3 > blast.bed  #  #  #use bedfile to obtain putative-hit fasta sequences, ignoring the errors into the “maybe_seq.err” file  bedtools getfasta -fi "$iso".fna -bed blast.bed -s > maybe_seq.fasta 2> maybe_seq.err  #  #first fix up the fasta file so that transeq will be okay with it…  cat maybe_seq.fasta \| sed 's/:/__/' > temp  #  #then translate into all reading frames  transeq -sequence temp -outseq temp_prot.fasta -frame F  #  #place each sequence onto its own line  cat temp_prot.fasta \| sed 's/__/:/' \| sed '/^>/ s/$/zzz/' \|tr -d '\n' \| sed $'s/zzz/\\\n/g' \| sed $'s/>/\\\n>/g' \| grep . > "$iso"_maybe_prot.fasta  #  #tidy  mv "$iso"_maybe_prot.fasta ./putative_msdin_prots/  mv "$iso"_chrm_name_key ./putative_msdin_prots/  #  rm "$iso".fna "$iso".fna.fai "$iso".fna.ndb "$iso".fna.nhr "$iso".fna.nin "$iso".fna.not "$iso".fna.nsq "$iso".fna.ntf "$iso".fna.nto  rm maybe_seq.err maybe_seq.fasta temp_prot.fasta blast_result chrm_lengths temp blast.bed temp_blast.bed  #  done < name_list  rm -r mast_output/ |
| MSDIN_motif.sh |
| #!/bin/bash  #########################################################################################  #FUNCTION  #  #This script uses motifs of MSDIN leader and CVGDD follower sequence to identify where in the blast-hits transmitted from last script the MSDIN may be  #exactly. Mast filters to e-values less than 100 which is a lenient cutoff. The script also determines the orientation of the hit and requires  # that THE MSDIN hit precedes the CVGDD hit in the sequence. Requires that sequence start with ATG/M. While the script could still output a sequence  #lacking a CVGDD hit, it will not output one without an MSDIN hit.  #  #REQUIRED PROGRAMS  # the script assumes that you have the MEME suit installed and needs a PATH to mast – also defined at the top of the script below.  #if MEME suit has already been added to PATH, then set mast path variable to ""  #script assumes you have Bedtools installed and in path.  #  #REQUIRED FILES  #This script assumes: you have pre-built a set of motif files for the MSDIN leader sequence and for the CVGDDD beginning of the follower sequence  #these two should both be put together into a file that is designated in the “path_to_motif_file” variable below. Note that the order of motifs in the #file matters a lot as if motif 2 occurs before motif 1 then it ignores it. Note that the files used in our publication are included in the example #dataset  #  #REQUIRED FORMATTING OF INPUT  #script assumes output of previous script was output correctly and into the default folder  #the script again assumes that genomes are named “name in name list”.fna.gz and are present in a folder ./genomes/ although the input folder can be #modified at the top of the script.  #  #This script requires: a name of accessions like the previous script – single column and called “name_list”  #script requires definition of path to “maybe_prot” files generated in previous script (msdin_blast_new.sh) – should be fine if you have not changed the default.  #  #MISC  #script will self-tidy and while I’ve tried to avoid this, you shouldn’t store any important files in the run directory as they could be remove. All #files that need to be brought in need their paths specified at the top of the script  #  #########################################################################################  #  #Variables that may need defining ----------------------------------------------------------------  Genome_folder_path="./genomes/"  Path_to_mast="/Users/meme/bin/"  Path_to_motif_file="./final_motif.txt"  Path_to_maybe_prot="./putative_msdin_prots/"  #--------------------------------------------------------------------------------------------------------------------------------  #  mkdir deduped_and_gene  #  while read -r iso; do  #  # Current_time=`date +"%T"`  echo ===============================================  echo Beginning msdin_motif of $iso at $Current_time  echo ===============================================  #  #  #preprocess genome to make naming scheme appropriate for downstream use…  #bring in and unzip genome  cp "$Genome_folder_path""$iso".fna.gz .  gunzip "$iso".fna.gz  #  #for consistency with above parts of the pipeline – remove period that break first script  Working_iso=`echo $iso \| sed 's/\./_/g'`  #  #rename chromosomes and add chrm length to them…  #first rename file to have the header iso_chrm#  cat "$iso".fna \| awk -v iso="$Working_iso" -v chrm="0" '{if ($0 ~ ">") {print ">"iso"_chrm"++chrm} else {print}}' >temp.fna  #then create afile of chrm lengths to use in renaming where length is added to name  cat temp.fna \| awk '$0 ~ ">" {if (NR > 1) {print c;} c=0;printf substr($0,2,100) "\t"; } $0 !~ ">" {c+=length($0);} END { print c; }' \| awk '{print ">"$1,">"$1"_""length_"$2}' > temp_chrm_lengths  #  #add lengths to name  awk 'FNR==NR{a[$1]=$2;next} {if ($1 in a){$1=a[$1]}; print $0}' temp_chrm_lengths temp.fna > genome_with_lengths  mv genome_with_lengths "$iso".fna  rm temp.fna temp_chrm_lengths  #  cp "$Path_to_maybe_prot""$iso""_maybe_prot.fasta" .  # # #run mast motif search  "$Path_to_mast"mast "$Path_to_motif_file" "$iso""_maybe_prot.fasta" -oc mast_output -ev 100  grep 'sequence db=' ./mast_output/mast.xml \| sed 's/.*name=\"//' \| sed 's/\".*//'\| grep . > temp_names  #  #  while read;do  pval=`sed -n -e "/$REPLY/,/\/sequence/ p" ./mast_output/mast.xml \| grep combined_pvalue \| sed 's/.*pvalue=\"//' \| sed 's/\".*//'`  eval=`sed -n -e "/$REPLY/,/\/sequence/ p" ./mast_output/mast.xml \| grep combined_pvalue \| sed 's/.*evalue=\"//' \| sed 's/\".*//'`  #  Motif1_pos=`sed -n -e "/$REPLY/,/\/sequence/ p" ./mast_output/mast.xml \| grep 'idx="0"' \| head -1 \| sed 's/.*pos=\"//' \| sed 's/\".*//'`  Motif1_map=`sed -n -e "/$REPLY/,/\/sequence/ p" ./mast_output/mast.xml \| grep 'idx="0"' \| head -1 \| sed 's/.*match=\"//' \| sed 's/\".*//' \| sed 's/^/\|/' \| sed 's/$/\|/'`  #  Motif2_pos=`sed -n -e "/$REPLY/,/\/sequence/ p" ./mast_output/mast.xml \| grep 'idx="1"'\| head -1 \| sed 's/.*pos=\"//' \| sed 's/\".*//'`  Motif2_map=`sed -n -e "/$REPLY/,/\/sequence/ p" ./mast_output/mast.xml \| grep 'idx="1"' \| head -1 \| sed 's/.*match=\"//' \| sed 's/\".*//' \| sed 's/^/\|/' \| sed 's/$/\|/' `  #  echo -e $REPLY"\t"$pval"\t"$eval"\t"$Motif1_pos"\t"$Motif1_map"\t"$Motif2_pos"\t"$Motif2_map \| sed -E $'s/\)_/\)\t/'  done < temp_names > temp  #  #make sure MSDIN hit is before CVGDD hit (if there are indeed both hits) – reject if no MSDIN hit but not if missing CV hit  awk -F'\t' '$5!="" {print}' temp \| awk -F'\t' '{if ($7=="") {print} else if ($5<$7) {print}}'> processed_mast  #  # #the following trims the sequence based on hits above – orienting based on starting M as determined by MSDIN motif hit  while IFS=$'\t' read -r name frame pvalue evalue motif1_hit motif1_map motif2_hit motif2_map;do  #  #this is the in-file name not the name of a file  file_name=`echo $name"_"$frame`  seq=`grep -A 1 $file_name "$iso""_maybe_prot.fasta"\| tail -1`  length=`echo $name \| sed 's/.*length_//'\| sed 's/_.*//'\| sed 's/:.*//'`  Chrm=`echo $name \| sed 's/:.*//'`  #  Og_start=`echo $name \| sed 's/.*://' \| sed 's/-/ /' \| sed -E 's/\(/ /' \|sed -E 's/\)//' \| awk '{print $1}'`  Og_end=`echo $name \| sed 's/.*://' \| sed 's/-/ /' \| sed -E 's/\(/ /' \|sed -E 's/\)//' \| awk '{print $2}'`  Orient=`echo $name \| sed 's/.*://' \| sed 's/-/ /' \| sed -E 's/\(/ /' \|sed -E 's/\)//' \| awk '{print $3}'`  Orient_num=`echo $name \| grep +\| wc -l \|sed 's/ //g' `  #  #  Motif1_offset=`echo $motif1_map \| sed 's/\|//g' \| sed 's/+.*//'\| wc -c \| sed 's/ //g' \| awk -v og_offset="$motif1_hit" -v frame=$frame '{print ((og_offset)*3)}' \| awk -v orientation=$Orient -v frame=$frame '{if (orientation=="+") {print ($1-4)+frame} else {print ($1-4)+frame}}'`  Motif2_offset=`echo $motif2_map \| sed 's/\|//g' \| sed 's/+.*//'\| wc -c \| sed 's/ //g' \| awk -v og_offset="$motif2_hit" -v frame=$frame '{print ((og_offset)*3)+frame}'`  #  Motif1_offset_AA=` echo $motif1_map \| sed 's/\|//g' \| sed 's/+.*//'\| wc -c \| sed 's/ //g' \| awk -v og_offset="$motif1_hit" -v frame=$frame '{print (og_offset)}'`  Motif2_offset_AA=` echo $motif2_map \| sed 's/\|//g' \| sed 's/+.*//'\| wc -c \| sed 's/ //g' \| awk -v og_offset="$motif2_hit" -v frame=$frame '{print (og_offset)}'`  #  Trimmed_Seq=`echo $seq\| cut -c"$Motif1_offset_AA"-9999`  #  M_check1=`echo $Motif1_offset_AA \| awk '{print $1-1}' \| awk '{if ($1=="0") {print "1"} else {print}}'`  M_check2=`echo $Motif1_offset_AA \| awk '{print $1}' \| awk '{if ($1=="0") {print "1"} else {print}}'`  M_check3=`echo $Motif1_offset_AA \| awk '{print $1+1}' \| awk '{if ($1=="0") {print "1"} else {print}}'`  #  Is_previous_M=`echo $seq \| cut -c"$M_check1"-"$M_check1" \| awk '{if ($1=="M") {print "1"} else {print "0"}}'`  Is_current_M=`echo $seq \| cut -c"$M_check2"-"$M_check2" \| awk '{if ($1=="M") {print "1"} else {print "0"}}'`  Is_next_M=`echo $seq \| cut -c"$M_check3"-"$M_check3" \| awk '{if ($1=="M") {print "1"} else {print "0"}}'`  #  #process forward and reverse differently…  if [ $Orient_num -gt 0 ];then  #  #  #check if first value is M, if not look at one in, and then one before change the start value accordingly.  New_start_temp=`echo $Og_start\|awk -v frame=$frame -v offset="$Motif1_offset" -v check1=$Is_previous_M -v check2=$Is_current_M -v check3=$Is_next_M '{if (check2=="1") {print ($1+offset), "check2"} else if (check3=="1") { print ($1+offset+3),"check3"} else if (check1=="1") {print ($1+offset-3),"check1"} else if (check1=="0" && check2=="0" && check3=="0") {print ($1+offset), "NOCHECK"}}'`  #  New_start=`echo $New_start_temp \| awk '{print $1}'`  Check_lvl=` echo $New_start_temp \| awk '{print $2}'`  #  #if a nearby M was found then shift the sequence to show that  if [[ $Check_lvl != "check2" && $Check_lvl != "NOCHECK" ]];then  seq_shift=`echo $Check_lvl \| sed 's/check//' \| awk -v og_offset=$Motif1_offset_AA '{print ($1-2)+og_offset}'`  #  Trimmed_Seq=`echo $seq\| cut -c"$seq_shift"-9999`  fi  #  #New_start=`echo $Og_start \| awk -v offset="$Motif1_offset" '{print $1+offset}'`  New_end=`echo $New_start\| awk '{print $1+300}'`  echo -e $Chrm"\t"$New_start"\t"$New_end"\t"$Trimmed_Seq"__""$Motif1_offset_AA""__""$Check_lvl""__""$Is_previous_M""$Is_current_M""$Is_next_M""__""$Motif2_offset_AA""\t"$length"\t"$Orient \| awk -F'\t' '{if ($2<0) {$2="1"} {print $1"\t"$2"\t"$3"\t"$4"\t"$5"\t"$6}}' \| awk -F'\t' '{if ($3>$5) {$3=$5} {print $1"\t"$2"\t"$3"\t"$4"\t"$5"\t"$6}}'  #  else  #  New_end_temp=`echo $Og_end \| awk -v frame=$frame -v offset="$Motif1_offset" -v check1=$Is_previous_M -v check2=$Is_current_M -v check3=$Is_next_M '{if (check2=="1") {print ($1-offset), "check2"} else if (check3=="1") { print ($1-offset-3),"check3"} else if (check1=="1") {print ($1-offset+3),"check1"} else if (check1=="0" && check2=="0" && check3=="0") {print ($1+offset), "NOCHECK"}}'`  #  New_end=`echo $New_end_temp \| awk '{print $1}'`  Check_lvl=`echo $New_end_temp \| awk '{print $2}'`  #  if [[ $Check_lvl != "check2" && $Check_lvl != "NOCHECK" ]];then  seq_shift=`echo $Check_lvl \| sed 's/check//' \| awk -v og_offset=$Motif1_offset_AA '{print ($1-2)+og_offset}'`  #  Trimmed_Seq=`echo $seq\| cut -c"$seq_shift"-9999`  fi  #  #New_end=`echo $Og_end \| awk -v offset="$Motif1_offset" -v frame=$frame '{print $1-offset+3-frame}'`  New_start=`echo $New_end\| awk '{print $1-300}'`  #  echo -e $Chrm"\t"$New_start"\t"$New_end"\t"$Trimmed_Seq"__""$Motif1_offset_AA""__""$Check_lvl""__""$Is_previous_M""$Is_current_M""$Is_next_M""__""$Motif2_offset_AA""\t"$length"\t"$Orient \| awk -F'\t' '{if ($2<0) {$2="1"} {print $1"\t"$2"\t"$3"\t"$4"\t"$5"\t"$6}}' \| awk -F'\t' '{if ($3>$5) {$3=$5} {print $1"\t"$2"\t"$3"\t"$4"\t"$5"\t"$6}}'  #  fi  done < processed_mast > temp  #note that processed.bed may contain a truncated version of the protein relative to the bed file.. it just hasn’t been updated..  sort -u -k1,3 -k6,6 temp \| grep -v NOCHECK > processed.bed  #  #  #process a bed file for later use in the make_msdin_bed thing…  # cat processed.bed \| awk '{print $1,$2,$3,$1":"$2"-"$3"("$6")",$5,$6}' > "$iso"_msdin_deduped  # #  bedtools getfasta -fi "$iso".fna -bed processed.bed -s > probably_seq.fasta 2> maybe_seq.err  #  cat probably_seq.fasta \| sed '/^>/ s/$/zzz/' \| tr -d '\n' \| sed $'s/zzz/\t/g' \| sed $'s/>/\\\n>/g' \| grep . \| awk -v iso_file="$iso""_msdin_deduped" '{print $1,$2,iso_file}'> "$iso"_gene_file  #  #some of these are probably not necessary –  #The gene_file gets used in the next script, and deduped file gets used in make_msdin_bed_and_parse_motif.sh  #  mv "$iso"_gene_file ./deduped_and_gene/  mv "$iso"_msdin_deduped ./deduped_and_gene/  #  rm "$iso".fna "$iso".fna.fai "$iso"_maybe_prot.fasta  #  rm temp_names processed_mast temp processed.bed probably_seq.fasta maybe_seq.err  done < name_list |
| MSDIN_motif_process_runmodes.sh |
| #!/bin/bash  #########################################################################################  #FUNCTION  #This script will take prior information about what MSDINs look like and use them to find introns and output all possible intron combinations at a #given locus. The real goal of this script is to determine where the end of MSDINs may be. Both canonical and non-canonical introns will be considered.  #  #This script is designed to find the introns in the putative MSDIN sequences. The sequences that do not have putative introns in them are thus #eliminated as all known MSDINs have conserved intron structure. The MSDIN sequences where putative introns can be found will be processed and placed  #into a file called “where_is_stop” as, with the intron structure, the script can look for where a putative stop codon would be.  #all of the input sequences will be translated into protein and placed into a file called “all_unprocessed_prots.fasta”  #  #REQUIRED PROGRAMS  #This script assumes you have EMBOSS installed and in path  #  #REQUIRED INPUTS  #script assumes output of previous script was output correctly and into the default folder  #  #MISC  #this script allows: you to set the MSDIN leader/core/follower length ranges as well as the intron length range (defaults are already in place)  #  #OUTPUT  #all of the input sequences that did not have putative introns will be placed into a file called “unprocessed_prots.fasta”  #these could be used to look for pseudogenized MSDINs and or to look for MSDINs where current intron parameters erroneously eliminate/manipulate #sequences.  #all results will be placed into a folder called “preprocessed_msdins”  #########################################################################################  #  #Variables that may need defining ----------------------------------------------------------------  #following lengths are in AA  Leader_length_range="9-10"  core_length_range="6-10"  follower_length_range="15-19"  #following length in bp  intron_length_range="52-58"  gene_file_path="./deduped_and_gene/"  #  #the following variable defines how many codons back from the end of the follower to being looking for intron end.  follower_codon_intron_end="4"  #  #runmodes = "**proline_guided**" (requires proline at end of core – fastest and what was used in the manuscript) "**allow_no_proline**" (if core seqs don’t #have proline at end then test all #possible core ends – can be much slower – if prolines exist it will default to looking at them) "always_check" #(looks for all possible MSDINs even if #prolines are present in a position that suggests the end of core – #slowest option)  runmode="proline_guided"  #--------------------------------------------------------------------------------------------------------  #  #  cat "$gene_file_path"*gene_file \| awk '{print $1,toupper($2),$3}' > gene_file  #  Leader_start=`echo $Leader_length_range \| sed 's/-/ /' \| awk '{print $1}'`  Leader_end=`echo $Leader_length_range \| sed 's/-/ /' \| awk '{print $2}'`  #  Length_of_file=`cat gene_file \| wc -l \| sed 's/ //g' `  Length_of_file_multiplier=`seq $Leader_start $Leader_end \| wc -l \| sed 's/ //g'`  Final_length_of_process=`echo $Length_of_file $Length_of_file_multiplier \| awk '{print $1*$2}'`  #  COUNTER="1"  #  rm where_is_stop  #  for Leader_length in $(seq $Leader_start $Leader_end);do  #  #we can ignore anything before this range hence removing it over and over below.. +1 because it starts one after the leader  Leader_nuc_len=`echo $Leader_length \| awk '{print $1*3}'`  Leader_nuc_len_plus_one=`echo $Leader_length \| awk '{print ($1*3)+1}'`  Def_core=`echo $core_length_range \| sed 's/-/ /' \| awk -v leader=$Leader_nuc_len '{print ($1-1)*3+leader}'`  start_core=`echo $core_length_range \| sed 's/-/ /' \| awk -v leader_plus_one=$Leader_nuc_len_plus_one '{print ($1-1)*3+leader_plus_one}'`  end_core=`echo $core_length_range \| sed 's/-/ /' \| awk -v leader=$Leader_nuc_len '{print $2*3+leader}'`  #adding one back in seems to deal with it cutting the core region short by one.. again may have to do with how "cut" is inclusive or not.  core_range=` echo $end_core $start_core \| sed 's/-/ /' \| awk '{print $1-$2+1}'`  maybe_proline_range=`echo $core_range $Def_core \| awk '{print $2-$1}'`  Def_coreAA=`echo $core_length_range \| sed 's/-.*/ /' \| awk '{print ($1-1)}'`  #  #according to pulman the intron always occurs in the fourth-to-last amino acid (including stop) so that's where we'll start looking..  start_follower=`echo $follower_length_range \| sed 's/-/ /' \| awk -v intron_end_codon="$follower_codon_intron_end" '{print ($1-intron_end_codon)*3}'`  end_follower=`echo $follower_length_range \| sed 's/-/ /' \| awk '{print $2*3}'`  #  intron_low=`echo $intron_length_range \| sed 's/-/ /' \| awk '{print $1}'`  intron_high=`echo $intron_length_range \| sed 's/-/ /' \| awk '{print $2}'`  #  echo $Leader_length $start_core $end_core $core_range  echo $Leader_length $start_follower $end_follower  #  #Name of file is just transmitted to resulting maps and is not used here.  while read gene_name sequence FILE;do  #  echo ----------------------------------------------------------------  echo starting line $COUNTER of $Final_length_of_process  echo ----------------------------------------------------------------  #  COUNTER=$[COUNTER + 1]  #  LEADER_SEQUENCE=`echo $sequence\| cut -c1-"$Leader_nuc_len"`  DEFINITELY_CORE_SEQUENCE=`echo $sequence\| cut -c"$Leader_nuc_len_plus_one"-"$Def_core" `  MAYBE_CORE_SEQUENCE=`echo $sequence\| cut -c"$start_core"-"$end_core" `  #  #the above assumes that the length of the core is at least 6AA without the P so the following will look for the end of the core at AA 7-10  #  #remove stuff before one would expect to find a P in the core range and then look for Ps afterwards. Mark these as possible end of core.  #output indicates which codon in the range is a P and COULD be the end of the core. If there are multiple it should iterate through them…  #this should be cutting it in a way that the ‘definitely core’ is not included – this is based off of the core length defined above.  #  if [[ $runmode =~ (proline_guided\|allow_no_proline) ]];then  echo $sequence\| cut -c"$start_core"-9999 \| cut -c1-"$core_range" \|sed $'s/.\{3\}/&\\\n/g' \| grep -n '\(CCT\|CCC\|CCA\|CCG\)' \| sed 's/:.*//' > possible_end_of_core  fi  #  #if end of core does not have a proline then just look at the last few codons in the possible core region  #  if [[ $runmode =~ (allow_no_proline\|always_check) ]];then  Possible_end_of_core_check=`cat possible_end_of_core \| grep . \| wc -l \| sed 's/ //g' `  if [ $Possible_end_of_core_check -eq 0 ];then  echo no proline found in range  #  Leader_minAA=`echo $Leader_length_range \| sed 's/-.*//'`  Core_minAA=` echo $core_length_range \| sed 's/-.*//' `  Leader_maxAA=`echo $Leader_length_range \| sed 's/.*-//'`  Core_maxAA=` echo $core_length_range \| sed 's/.*-//' `  #  #  #  end_coresearch_manual=`echo $Def_coreAA $Core_maxAA \| awk ' {print $2-$1}' `  for x in $( eval echo {1..$end_coresearch_manual} );do echo $x ;done >possible_end_of_core  else  echo proline found  fi  fi  #  while read end_core_proline_codon;do  #now we start looking from the proline to look for intron start/end cites  #  #  #at this point counting is done relative to the leader+def core  Earliest_intron_start=` echo $end_core_proline_codon\| awk -v start_follower="$start_follower" -v core="$start_core" '{print $1*3+start_follower+core+1}'`  Latest_intron_start=` echo $end_core_proline_codon\| awk -v end_follower="$end_follower" -v core="$start_core" '{print $1*3+end_follower+core}'`  #  #look for potential start of the intron  MAYBE_INTRON_START=` echo $sequence \| cut -c"$Earliest_intron_start""-""$Latest_intron_start" `  #  echo $sequence \| cut -c"$Earliest_intron_start"-"$Latest_intron_start" \|sed $'s/GT/&\\\n/g' \| grep GT\| awk '{ print $1, length-2 }'> possible_intron_start  echo $sequence \| cut -c"$Earliest_intron_start"-"$Latest_intron_start" \|sed $'s/GC/&\\\n/g' \| grep GC\| awk '{ print $1, length-2 }'> non_canon_possible_intron_start  #  #  Current_location="0"  Loop="0"  while read ignore_sequence possible_intron_start;do  #  if [ $Loop -gt 0 ];then  Current_location=$[ Current_location + 2 + possible_intron_start ]  else  Current_location=$[ Current_location + possible_intron_start ]  fi  #  #  Earliest_intron_end=`echo 0 \| awk -v start="$Earliest_intron_start" -v intron_low="$intron_low" '{print $1+start+intron_low}'`  Latest_intron_end=` echo 0 \| awk -v start="$Latest_intron_start" -v intron_high="$intron_high" '{print $1+start+intron_high}'`  #  MAYBE_INTRON_END=` echo $sequence \| cut -c"$Earliest_intron_end"-"$Latest_intron_end" `  #  #  echo $sequence\| cut -c"$Earliest_intron_end"-"$Latest_intron_end" \| grep 'AG' \| wc -l \| sed 's/ //g'> possible_intron_end_codons  #  #determine if that are ANY GTs that could end the intron and if so then move forward  Any_ends=`cat possible_intron_end_codons`  #  Loop=$[ Loop + 1]  #  if [ $Any_ends -gt 0 ];then  #  echo $sequence \| cut -c"$Earliest_intron_end"-"$Latest_intron_end" \| sed $'s/AG/AG\\\n/g' \| grep AG \| awk '{ print length }' \| tr '\n' ' '\| awk '{for (i = 1; i <= NF; ++i) {printf("%d ", s += $i);} printf("\n");}' \| tr ' ' '\n' \| grep . > possible_ends  #  #loop through accounting for the length of the GT and where the new reading frame would start… -- still haven't vetted these numbers for current frame  #  #  while read end;do  current_frame=`echo $Earliest_intron_end \| awk -v value=$end '{print $1+value+4"-"$1+value+8+1}'`  #  current_frame_codon=`echo $sequence \| cut -c"$current_frame" \| grep '\(TAA\|TAG\|TGA\)'\| sed 's/ //g'\| wc -l`  end_seq=` echo $sequence \| cut -c"$current_frame"`  #  MAYBE_STOP=` echo $sequence \| cut -c"$current_frame" `  #  Last_bp_of_exon1=`echo $Earliest_intron_start \| awk -v current=$Current_location '{print $1+current-1}'`  Start_of_exon2=`echo "$Earliest_intron_end"\| awk -v end=$end '{print $1+end}'`  End_of_exon2=`echo $current_frame \| sed 's/-/ /'\| awk '{print $2}'`  #  SEQUENCE_AFTER_DEF_CORE=`echo $sequence \| cut -c"$start_core"-"$Last_bp_of_exon1"`  SEQUENCE_AFTER_INTRON=`echo $sequence \| cut -c"$Start_of_exon2"-"$End_of_exon2"`  #  echo $possible_intron_start $start_core $start_follower $end_core_proline_codon $Earliest_intron_start $Latest_intron_start "$Earliest_intron_end" "$Latest_intron_end" $Earliest_intron_start $Last_bp_of_exon1 $Start_of_exon2 $End_of_exon2 $end_core $core_range  #  CDS=`echo "$LEADER_SEQUENCE""$DEFINITELY_CORE_SEQUENCE""$SEQUENCE_AFTER_DEF_CORE""$SEQUENCE_AFTER_INTRON"`  #  echo ">really_dumb_that_transeq_needs_header" > temp_seq  echo $CDS >> temp_seq  transeq -sequence temp_seq -outseq temp_out -sformat pearson 2> silencing_transeq_just_saying_it_has_translated_stuff  rm silencing_transeq_just_saying_it_has_translated_stuff  #  cat temp_out\| sed '/^>/ s/$/zzz/' \|tr -d '\n' \| sed $'s/zzz/\\\n/g' \| sed $'s/>/\\\n>/g' \| grep . > ugh  mv ugh temp_out  #  PROTEIN=`cat temp_out \| tail -1`  rm temp_seq temp_out  #  echo "$gene_name"stop_search_"$end_core_proline_codon"_"$Current_location"_"$end" $current_frame $Last_bp_of_exon1 $Start_of_exon2 $End_of_exon2 $Leader_length $Def_core $current_frame_codon $end_seq $LEADER_SEQUENCE $DEFINITELY_CORE_SEQUENCE $MAYBE_CORE_SEQUENCE $MAYBE_INTRON_START $MAYBE_INTRON_END $MAYBE_STOP $CDS $PROTEIN $FILE  #  done < possible_ends >> where_is_stop  #  echo Current output DOES include stop in range  #  else  echo Current output DOES NOT include stop in range  fi  #  #this will be needed to shift us forward from the last possible start location.. this is mostly for intron searching  #  done < possible_intron_start  #  #  #  #  #  #  #The following is the same as above but it will process non-canonical introns  Current_location="0"  Loop="0"  while read ignore_sequence possible_intron_start;do  #  if [ $Loop -gt 0 ];then  Current_location=$[ Current_location + 2 + possible_intron_start ]  else  Current_location=$[ Current_location + possible_intron_start ]  fi  #  #  Earliest_intron_end=`echo 0 \| awk -v start="$Earliest_intron_start" -v intron_low="$intron_low" '{print $1+start+intron_low}'`  Latest_intron_end=` echo 0 \| awk -v start="$Latest_intron_start" -v intron_high="$intron_high" '{print $1+start+intron_high}'`  #  MAYBE_INTRON_END=` echo $sequence \| cut -c"$Earliest_intron_end"-"$Latest_intron_end" `  #  #  echo $sequence\| cut -c"$Earliest_intron_end"-"$Latest_intron_end" \| grep 'AG' \| wc -l \| sed 's/ //g'> possible_intron_end_codons  #  #determine if that are ANY AGs that could end the intron and if so then move forward  Any_ends=`cat possible_intron_end_codons`  #  Loop=$[ Loop + 1]  #  if [ $Any_ends -gt 0 ];then  #  echo $sequence \| cut -c"$Earliest_intron_end"-"$Latest_intron_end" \| sed $'s/AG/AG\\\n/g' \| grep AG \| awk '{ print length }' \| tr '\n' ' '\| awk '{for (i = 1; i <= NF; ++i) {printf("%d ", s += $i);} printf("\n");}' \| tr ' ' '\n' \| grep . > possible_ends  #  #loop through accounting for the length of the AG and where the new reading frame would start… -- still aven't vetted these numbers for current frame  #  #  while read end;do  current_frame=`echo $Earliest_intron_end \| awk -v value=$end '{print $1+value+4"-"$1+value+8+1}'`  #  current_frame_codon=`echo $sequence \| cut -c"$current_frame" \| grep '\(TAA\|TAG\|TGA\)'\| sed 's/ //g'\| wc -l`  end_seq=` echo $sequence \| cut -c"$current_frame"`  #  MAYBE_STOP=` echo $sequence \| cut -c"$current_frame" `  #  Last_bp_of_exon1=`echo $Earliest_intron_start \| awk -v current=$Current_location '{print $1+current-1}'`  Start_of_exon2=`echo "$Earliest_intron_end"\| awk -v end=$end '{print $1+end}'`  End_of_exon2=`echo $current_frame \| sed 's/-/ /'\| awk '{print $2}'`  #  SEQUENCE_AFTER_DEF_CORE=`echo $sequence \| cut -c"$start_core"-"$Last_bp_of_exon1"`  SEQUENCE_AFTER_INTRON=`echo $sequence \| cut -c"$Start_of_exon2"-"$End_of_exon2"`  #  echo $possible_intron_start $start_core $start_follower $end_core_proline_codon $Earliest_intron_start $Latest_intron_start "$Earliest_intron_end" "$Latest_intron_end" $Earliest_intron_start $Last_bp_of_exon1 $Start_of_exon2 $End_of_exon2 $start_core  #  CDS=`echo "$LEADER_SEQUENCE""$DEFINITELY_CORE_SEQUENCE""$SEQUENCE_AFTER_DEF_CORE""$SEQUENCE_AFTER_INTRON"`  #  echo ">really_dumb_that_transeq_needs_header" > temp_seq  echo $CDS >> temp_seq  transeq -sequence temp_seq -outseq temp_out -sformat pearson 2> silencing_transeq_just_saying_it_has_translated_stuff  rm silencing_transeq_just_saying_it_has_translated_stuff  #  cat temp_out\| sed '/^>/ s/$/zzz/' \|tr -d '\n' \| sed $'s/zzz/\\\n/g' \| sed $'s/>/\\\n>/g' \| grep . > ugh  mv ugh temp_out  #  PROTEIN=`cat temp_out \| tail -1`  rm temp_seq temp_out  #  echo "$gene_name"stop_search_"$end_core_proline_codon"_"NC""$Current_location"_"$end" $current_frame $Last_bp_of_exon1 $Start_of_exon2 $End_of_exon2 $Leader_length $Def_core $current_frame_codon $end_seq $LEADER_SEQUENCE $DEFINITELY_CORE_SEQUENCE $MAYBE_CORE_SEQUENCE $MAYBE_INTRON_START $MAYBE_INTRON_END $MAYBE_STOP $CDS $PROTEIN $FILE  #  done < possible_ends >> where_is_stop  #  echo Current output DOES include stop in range  #  else  echo Current output DOES NOT include stop in range  fi  #  #this will be needed to shift us forward from the last possible start location.. this is mostly for intron searching  #  done < non_canon_possible_intron_start  #  done < possible_end_of_core  #  rm possible_end_of_core  #  done < gene_file  done  #  cat gene_file \| awk '{print $1,$2}'\| sed 's/>//'\| sed 's/^/>/' \| tr '\t' '\n'\| tr ' ' '\n' > all_unprocessed_cds  transeq -sequence all_unprocessed_cds -outseq all_unprocessed_prots.fasta -sformat pearson 2> silencing_transeq_just_saying_it_has_translated_stuff  rm silencing_transeq_just_saying_it_has_translated_stuff  #  cat all_unprocessed_prots.fasta \| sed '/^>/ s/$/zzz/' \|tr -d '\n' \| sed $'s/zzz/\\\n/g' \| sed $'s/>/\\\n>/g' \| grep . > ugh  mv ugh all_unprocessed_prots.fasta  #  rm all_unprocessed_cds possible_ends possible_intron_end_codons possible_intron_start possible_end_of_core non_canon_possible_intron_start possible_intron_codons possible_intron_start_codons  #  cat where_is_stop \| grep '>' \| sort -u > temp  mv temp where_is_stop  #  #get list of all processed/unprocessed hits  awk '{print $1}' where_is_stop \| sed 's/stop_search.*//'\|sort -u > processed_hits  awk '{print $1}' gene_file \|sort -u >all_hits  cat all_hits processed_hits \| sort \| uniq -u > unprocessed_hits  #  awk 'NR==FNR{c[$1]++;next};c[$1] > 0' unprocessed_hits gene_file \| awk '{print $1"\n"$2}' > unprocessed_genes.fasta  transeq -sequence unprocessed_genes.fasta -outseq temp_out -sformat pearson 2> silencing_transeq_just_saying_it_has_translated_stuff  rm silencing_transeq_just_saying_it_has_translated_stuff  #  cat temp_out\| sed '/^>/ s/$/zzz/' \|tr -d '\n' \| sed $'s/zzz/\\\n/g' \| sed $'s/>/\\\n>/g' \| grep . > ugh  mv ugh unprocessed_prots.fasta  #  rm unprocessed_genes.fasta processed_hits all_hits unprocessed_hits  #  mkdir preprocessed_msdins  mv where_is_stop ./preprocessed_msdins/  mv unprocessed_prots.fasta ./preprocessed_msdins/  mv all_unprocessed_prots.fasta ./preprocessed_msdins/ |
| make_msdin_bed_and_parse_motif.sh |
| #!/bin/bash  #########################################################################################  #FUNCTION  #This script will take the processed output of msdin_motif_process.sh – “where_is_stop” and will create a bed file for the entire msdin (including the #identified intron)while also extracting the leader, core and follower sequences. note that finalized beds for best sequences are generated later.  #  #the script will first filter the output of where_is_stop to include only MSDIN sequences that actually end in a ‘stop’.  #It will then filter sequences to include only those with a single stop codon  #  #REQUIRED INPUT  #script assumes output of previous script was output correctly and into the default folder  #  #MISC  #I have had a little trouble getting different programs to interpret bed files the same way, some seem to be inclusive at both ends of a bed entry #while others are not thus, depending on what downstream application you’re using this for please confirm that the end of bed entries are not off by #+/- 1 – this should work correctly for programs used here.  #########################################################################################  #  #Variables that may need defining ----------------------------------------------------------------  Path_to_deduped="./deduped_and_gene/"  Path_to_where_is_stop="./preprocessed_msdins/"  # ------------------------------------------------------------------------------------------------------  #  #filter out sequences based ending in a stops/having too many stops.  cat "$Path_to_where_is_stop""where_is_stop" \| grep '\* ' \| awk '{print $1,$17,$18}' \| sed 's/stop_search/ stop_search/' \| awk -F'\*' '{print $0,NF-1}' \| awk -F'_NC' '{print $0,NF-1}' \| awk '$5=="1" {print}' \| sort -k4,4\| sed 's/>//' > processed_best_msdin  #  Number_of_msdins_to_process=`cat processed_best_msdin \| wc -l \| sed 's/ //g'`  COUNTER="1"  #  rm parsed_best_msdin processed_best_msdin.bed  while read old_name stop_search seq file scores;do  echo beginning to process msdin $COUNTER of $Number_of_msdins_to_process named: "$file" $old_name "prot"  #  #lets start by processing the protein into its constituents  #  Leader_end=` grep $old_name "$Path_to_where_is_stop"where_is_stop \| grep -w $stop_search \| awk -v seq="$seq" '$17==seq {print}'\|grep $file \| awk '{print $6}'`  Core_start=`echo $Leader_end \| awk '{print $1+1}'`  Core_end_proline=`echo $stop_search\| sed 's/stop_search_//' \| sed 's/_.*//'`  Core_end=` grep $old_name "$Path_to_where_is_stop"where_is_stop \| grep -w $stop_search \| awk -v seq="$seq" '$17==seq {print}'\|grep $file\| awk -v leader=$Leader_end -v end_proline=$Core_end_proline '{print ($7/3)+end_proline}'`  Follower_start=`echo $Core_end \| awk '{print $1+1}'`  #  Leader=`echo $seq \| cut -c1-"$Leader_end"`  Core=`echo $seq \| cut -c"$Core_start"-"$Core_end"`  Follower=` echo $seq \| cut -c"$Follower_start"-9999`  #  #echo $seq $Leader_end $Core_end_proline $Core_end  echo "$file" "PROT" $old_name"_z_""$stop_search""_x_" $Leader $Core $Follower $seq >> parsed_best_msdin  #  #  #Okay now lets generate the cds for these…  echo beginning to process msdin $COUNTER of $Number_of_msdins_to_process named: "$file" $old_name "nuc"  #  Full_cds=` grep $old_name "$Path_to_where_is_stop"where_is_stop \| grep -w $stop_search \| awk -v seq="$seq" '$17==seq {print}'\|grep $file \| awk '{print $16}'`  #  Nuc_intron_start=` echo $Core_end `  Nuc_leader_end=`echo $Leader_end\| awk '{print ($1*3)}'`  Nuc_core_start=`echo $Nuc_leader_end \| awk '{print $1+1}'`  Nuc_core_end=`echo $Core_end \| awk '{print $1*3}'`  Nuc_follower_start=`echo $Nuc_core_end \| awk '{print $1+1}'`  #  #  Nuc_leader=` echo $Full_cds \| cut -c1-"$Nuc_leader_end"`  Nuc_core=` echo $Full_cds \| cut -c"$Nuc_core_start"-"$Nuc_core_end"`  Nuc_follower=` echo $Full_cds \| cut -c"$Nuc_follower_start"-9999`  #  #echo $Full_cds $nuc_leader_end $Nuc_core_start $Nuc_core_end $Nuc_follower_start  echo "$file" "NUC" $old_name"_z_""$stop_search""_x_" $Nuc_leader $Nuc_core $Nuc_follower $Full_cds >> parsed_best_msdin  #  #okay now lets generate a bed file for them…  echo beginning to process msdin $COUNTER of $Number_of_msdins_to_process named: "$file" $old_name "bed"  COUNTER=$[COUNTER + 1]  #  Chrm=`grep $old_name "$Path_to_deduped""$file" \| awk '{print $1}'`  Orient=`grep $old_name "$Path_to_deduped""$file"\| awk '{print $6}'`  Og_low=`grep $old_name "$Path_to_deduped""$file"\| awk '{print $2}'`  Og_high=`grep $old_name "$Path_to_deduped""$file"\| awk '{print $3}'`  #  End_exon1=` grep $old_name "$Path_to_where_is_stop"where_is_stop \| grep -w $stop_search \| awk -v seq="$seq" '$17==seq {print}'\|grep $file \| awk '{print $3+1}'`  Start_intron=`echo $End_exon1 \| awk '{print $1+1}'`  #DELETED A PLUS ONE at end of following line for $4 initial testing seems to suggest this is correct  End_intron=` grep $old_name "$Path_to_where_is_stop"where_is_stop \| grep -w $stop_search \| awk -v seq="$seq" '$17==seq {print}'\|grep $file \| awk '{print $4}'`  #  Length=` echo $Full_cds \| wc -c \| sed 's/ //g' `  Exon_remaining=`echo $End_exon1 $Length \| awk '{print $2-$1}'`  End_exon2=`echo $End_intron $Exon_remaining\| awk '{print $1+$2}'`  #  echo $Chrm $Og_low $Og_high "exon1""_z_""$old_name""__""$file""_z_""$stop_search""_x_" "NA" $Orient \|awk -v end=$End_exon1 '{if ($6=="+") {print $1"\t"$2+1"\t"$2+end"\t"$4"\t"$5"\t"$6} else {print $1"\t"$3-end"\t"$3-1"\t"$4"\t"$5"\t"$6}}' > temp.bed  #  echo $Chrm $Og_low $Og_high "intron1""_z_""$old_name""__""$file""_z_""$stop_search""_x_" "NA" $Orient \|awk -v start=$Start_intron -v end=$End_intron '{if ($6=="+") {print $1"\t"$2+start-1"\t"$2+end"\t"$4"\t"$5"\t"$6} else {print $1"\t"$3-end"\t"$3-start+1"\t"$4"\t"$5"\t"$6}}' >> temp.bed  #  #DELETED A PLUS ONE at end of reverse end that initial testing supports  echo $Chrm $Og_low $Og_high "exon2""_z_""$old_name""__""$file""_z_""$stop_search""_x_" "NA" $Orient \|awk -v start=$End_intron -v end=$End_exon2 '{if ($6=="+") {print $1"\t"$2+start"\t"$2+end"\t"$4"\t"$5"\t"$6} else {print $1"\t"$3-end"\t"$3-start"\t"$4"\t"$5"\t"$6}}' >> temp.bed  #  cat temp.bed >> processed_best_msdin.bed  rm temp.bed  done < processed_best_msdin  #  mkdir processed_msdin  mv processed_best_msdin* ./processed_msdin/  mv parsed_best_msdin ./processed_msdin/ |
| MSDIN_score_and_locus.sh |
| #!/bin/bash  #########################################################################################  #FUNCTION  #This script will take the processed output of make_msdin_bed_and_parse_motif.sh and score the resulting possible MSDINs that were identified  # requires and updates the parsed_best_msdin and processed_best_msdin.bed files generated in previous  #the script will assign a single point for each of the following:  #one point per match for the exact motif “CVGDDV”  #thirty points if the core region has been identified previously  #one point each for the final L and C residues.  #one point if the length of the follower is between 16 and 18 inclusive  #one point if the leader ends in a P  #  #REQUIRED FILES  #script assumes that there is a file called “MSDIN_genes_all_lib.txt” that is tab delimited text with the columns: species, msdin_seq, toxin (if #known), msdin_leader_seq,msdin_core_seq,msdin_follower_seq,sorce_of_info – really its only important that the fifth column be the core sequence  #  #REQUIRED INPUT  #script assumes output of previous script was output correctly and into the default folder  #  #OUTPUT NOTES  #the script will generate and output the following files into  #a folder called “final_msdin”  #  #the script will output:  #  #parsed_best_msdin_prot_with_scores – all of the scored MSDINs (just the prots). This file’s columns include – note some column names are not defined #here because they’re not relevant to end users: 1) a name that includes the original name of the input genome (access for NCBI). 3) a naming scheme #that refers to where the MSDIN was found in the genome and a code that indicates how to process it. These names can be used to reference other files.  # 4) the protein leader sequence  # 5) the protein core sequence  # 6) the protein follower sequence  # 7) the full MSDIn sequence  #  #multiple_msdin_same_locus – a list of msdin loci that contain multiple of the same highest score msdins (this is rare and may be empty)  #  #one_per_locus_high_scoring – a subset of msdins from parsed_best_msdin_prot_with_scores where only the highest scoring msdin per locus is kept. If #multiple msdin have the same score at a given locus (rare) then one is chosen randomly – in my experience, such MSDINs do not pass final filtering  #  # one_per_locus_high_scoring_with_scores  #same information as one_per_locus_high_scoring but additionally a summed score is indicated in the last column  #  # one_per_locus_high_scoring_with_scores  #same information as one_per_locus_high_scoring_with_scores but has been filtered by score based on user-defined inputs  #  #parsed_best_msdin_final this file contains very similar information as parsed_best_msdin_prot_with_scores and one_per_locus_high_scoring but will also #contain the multiple highest scoring at the same locus – it also includes nucleotide info not just amino acid in a format similar to that of #parsed_best_msdin_prot_with_scores  #  #  #processed_best_msdin_final.bed this contains a bed file for all of the ones from one_per_locus_high_scoring naming in this file corresponds to that in #the third and fourth column of the one_per_locus_high_scoring file. Remember that chromosomes have been renamed, and that a key to the renaming scheme #is available in the putative_msdin_prots folder created by the first script.  #  #  #########################################################################################  #  ##Variables that may need defining ----------------------------------------------------------------  Path_to_parsed_best_msdin="./processed_msdin/"  Path_to_known_msdin_lib="./"  Score_to_filter_to="5"  #----------------------------------------------------------------  #  awk '$2=="PROT" {print $1,$7}' "$Path_to_parsed_best_msdin"parsed_best_msdin \| sort -u > temp  #  Count_of_msdin_to_score=`cat temp \| wc -l \| sed 's/ //g'`  COUNTER="1"  #  rm parsed_best_msdin_prot_with_scores  while read iso msdin;do  #  #  echo -------------------------------------------------  echo SCORING msdin $COUNTER of $Count_of_msdin_to_score  echo -------------------------------------------------  #  COUNTER=$[COUNTER + 1]  #  awk -v iso="$iso" -v msdin="$msdin" '$1==iso && $7==msdin {print}' "$Path_to_parsed_best_msdin"parsed_best_msdin > current_msdin  #  Current_count=`cat current_msdin \| wc -l \| sed 's/ //g'`  #  if [ $Current_count -gt 1 ];then  #  #lets score the different possible locations of the core and print them out..  #  while read -r name prot contig leader core follower full;do  #  Pos_1=`echo $follower \| sed 's/./& /g' \| awk '{if ($1=="C") {print "1"} else {print "0"} }'`  Pos_2=`echo $follower \| sed 's/./& /g' \| awk '{if ($2=="V") {print "1"} else {print "0"} }'`  Pos_3=`echo $follower \| sed 's/./& /g' \| awk '{if ($3=="G") {print "1"} else {print "0"} }'`  Pos_4=`echo $follower \| sed 's/./& /g' \| awk '{if ($4=="D") {print "1"} else {print "0"} }'`  Pos_5=`echo $follower \| sed 's/./& /g' \| awk '{if ($5=="D") {print "1"} else {print "0"} }'`  Pos_6=`echo $follower \| sed 's/./& /g' \| awk '{if ($6=="V") {print "1"} else {print "0"} }'`  #  Canon=`echo $contig \| grep _NC \| wc -l \| sed 's/ //g'`  #  #lets score if the MSDIN is known..  cat "$Path_to_known_msdin_lib"MSDIN_genes_all_lib.txt \| awk -F'\t' 'NR>1 {print $5}' \| sort -u > all_known_msdin  scoreKNOWN=`grep -w $core all_known_msdin\| head -1 \| wc -l \| sed 's/ //g' \| awk '{print $1*30}'`  #  #lets score the end of the protein too..  #  PosC=`echo $follower \| sed 's/./& /g' \| awk '{if ($(NF-1)=="C") {print "1"} else {print "0"} }'`  PosL=`echo $follower \| sed 's/./& /g' \| awk '{if ($(NF-2)=="L") {print "1"} else {print "0"} }'`  #  scoreLC=`echo -e "$PosL""\n""$PosC"\| awk '{sum+=$1} END {print sum}'`  score=`echo -e "$Pos_1""\n""$Pos_2""\n""$Pos_3""\n""$Pos_4""\n""$Pos_5""\n""$Pos_6" \| awk '{sum+=$1} END {print sum}'`  #  score_follower_length=` echo $follower \|sed 's/\*//'\| awk '{print length}'\|awk '{if ($1 >= 16 && $1<=18) {print "1"} else {print "0"}}' `  #  #okay and lets add a score for the leader too…  score_leader=`echo $leader \| sed 's/./& /g' \| awk '{if ($(NF)=="P") {print "1"} else {print "0"} }'`  #  echo $name $prot $contig $leader $core $follower $full $score $scoreLC $score_leader $score_follower_length $scoreKNOWN "contested" $Canon >> parsed_best_msdin_prot_with_scores  done < <(cat current_msdin)  #  else  #  while read -r name prot contig leader core follower full;do  #  Pos_1=`echo $follower \| sed 's/./& /g' \| awk '{if ($1=="C") {print "1"} else {print "0"} }'`  Pos_2=`echo $follower \| sed 's/./& /g' \| awk '{if ($2=="V") {print "1"} else {print "0"} }'`  Pos_3=`echo $follower \| sed 's/./& /g' \| awk '{if ($3=="G") {print "1"} else {print "0"} }'`  Pos_4=`echo $follower \| sed 's/./& /g' \| awk '{if ($4=="D") {print "1"} else {print "0"} }'`  Pos_5=`echo $follower \| sed 's/./& /g' \| awk '{if ($5=="D") {print "1"} else {print "0"} }'`  Pos_6=`echo $follower \| sed 's/./& /g' \| awk '{if ($6=="V") {print "1"} else {print "0"} }'`  #  Canon=`echo $contig \| grep _NC \| wc -l \| sed 's/ //g'`  #  #lets score if the MSDIN is known..  cat "$Path_to_known_msdin_lib"MSDIN_genes_all_lib.txt \| awk -F'\t' 'NR>1 {print $5}' \| sort -u > all_known_msdin  scoreKNOWN=`grep -w $core all_known_msdin\| head -1 \| wc -l \| sed 's/ //g' \| awk '{print $1*30}' `  #  #lets score the end of the protein too..  #  PosC=`echo $follower \| sed 's/./& /g' \| awk '{if ($(NF-1)=="C") {print "1"} else {print "0"} }'`  PosL=`echo $follower \| sed 's/./& /g' \| awk '{if ($(NF-2)=="L") {print "1"} else {print "0"} }'`  #  scoreLC=`echo -e "$PosL""\n""$PosC"\| awk '{sum+=$1} END {print sum}'`  score=`echo -e "$Pos_1""\n""$Pos_2""\n""$Pos_3""\n""$Pos_4""\n""$Pos_5""\n""$Pos_6" \| awk '{sum+=$1} END {print sum}'`  #  #lets add a score for the length of the follower… if it falls in the normal, inclusive, range of 16-18 (without stop) then give it a point if not then #none  #  score_follower_length=` echo $follower \| sed 's/\*//'\| awk '{print length}'\|awk '{if ($1 >= 16 && $1<=18) {print "1"} else {print "0"}}' `  #  #okay and lets add a score for the leader too…  score_leader=`echo $leader \| sed 's/./& /g' \| awk '{if ($(NF)=="P") {print "1"} else {print "0"} }'`  #  echo $name $prot $contig $leader $core $follower $full $score $scoreLC $score_leader $score_follower_length $scoreKNOWN "uncontested" $Canon >> parsed_best_msdin_prot_with_scores  done < <(cat current_msdin)  #  fi  #  done < <(cat temp)  rm temp current_msdin  #  #  #  echo -------------------------------------------------  echo filtering scores by locus  echo -------------------------------------------------  #  #filter high scoring ones at same locus..  #  cat parsed_best_msdin_prot_with_scores \| sed 's/_z_/ _z_/' \| sort -k3,3 -k13,13nr -k11,11nr -k10,10nr -k9,9nr -k12,12nr > temp_scores  cat temp_scores \| sort -u -k3,3 \| awk '{print $3,$9,$10,$11,$12,$13}' > temp_loop  #  #obtain highest scores at each locus  #score 1 is CVGDDV score  #score 2 is the LC score  #score 3 is the leader score  #score 4 is based on follower length  #score 5 is based on if the msdin is known..  while read locus score1 score2 score3 score4 score5;do  #take the line(s) that has the high-scoring scores for that locus  awk -v locus="$locus" -v score1="$score1" -v score2="$score2" -v score3="$score3" -v score4="$score4" -v score5="$score5" '$3==locus && $9==score1 && $10==score2 && $11==score3 && $12==score4 && $13==score5 {print}' temp_scores  done < temp_loop >high_scoring  rm temp_scores temp_loop  #  #  #output those that have multiple identical scores at same locus:  awk '{c[$3]++}END{ for (i in c) print i,c[i]}' high_scoring \| sort -k2,2nr \| awk '$2>1 {print}' > multiple_msdin_same_locus  #  #randomly select one per locus from those that overlap…  shuf high_scoring \| sort -u -k3,3 > one_per_locus_high_scoring  #  #  #filter parsed and bed file based on filtering from scoring: #  #  echo --------------------------------------------------------------------------------  echo outputting final parsed and bed file based on highest scoring msdin per locus  echo --------------------------------------------------------------------------------  #  #lets deal with the parsed file first  while read -r name prot contig stop_search leader core follower full follower_start_score follower_end_score contested canon;do  #  Combo_name=`echo "$contig""$stop_search"`  #  awk -v combo="$Combo_name" '$3==combo {print}' "$Path_to_parsed_best_msdin"parsed_best_msdin  done < high_scoring > parsed_best_msdin_final  #  #  #and now the bed file..  #  while read -r name prot contig stop_search leader core follower full follower_start_score follower_end_score contested canon;do  #  Combo_name_exon1=`echo "exon1_z_""$contig""__""$name""$stop_search"`  Combo_name_intron1=`echo "intron1_z_""$contig""__""$name""$stop_search"`  Combo_name_exon2=`echo "exon2_z_""$contig""__""$name""$stop_search"`  #  awk -v combo="$Combo_name_exon1" '$4==combo {print}' "$Path_to_parsed_best_msdin"processed_best_msdin.bed  awk -v combo="$Combo_name_exon2" '$4==combo {print}' "$Path_to_parsed_best_msdin"processed_best_msdin.bed  awk -v combo="$Combo_name_intron1" '$4==combo {print}' "$Path_to_parsed_best_msdin"processed_best_msdin.bed  #  done < one_per_locus_high_scoring > processed_best_msdin_final.bed  #  cat one_per_locus_high_scoring \| awk '{{score_sum=$9+$10+$11+$12+$13} {print $0,score_sum}}' \| sort -k1,1 -k16,16nr > one_per_locus_high_scoring_with_scores  #  awk -v score="$Score_to_filter_to" '$16>=score {print}' one_per_locus_high_scoring_with_scores > one_per_locus_high_scoring_with_scores_filtered  #  mkdir final_msdin  mv parsed_best_msdin_prot_with_scores ./final_msdin  mv processed_best_msdin_final.bed ./final_msdin/  mv parsed_best_msdin_final ./final_msdin/  mv one_per_locus_high_scoring ./final_msdin/  mv multiple_msdin_same_locus ./final_msdin/  #  mv one_per_locus_high_scoring_with_scores ./final_msdin/  mv one_per_locus_high_scoring_with_scores_filtered ./final_msdin/ |
